# Highly multiplexed imaging reveals prognostic immune and stromal spatial biomarkers in breast cancer

**DOI:** 10.1172/jci.insight.176749

**Published:** 2025-01-14

**Authors:** Jennifer R. Eng, Elmar Bucher, Zhi Hu, Cameron R. Walker, Tyler Risom, Michael Angelo, Paula Gonzalez-Ericsson, Melinda E. Sanders, A. Bapsi Chakravarthy, Jennifer A. Pietenpol, Summer L. Gibbs, Rosalie C. Sears, Koei Chin

**Affiliations:** 1 Department of Molecular and Medical Genetics and; 2 Department of Biomedical Engineering, Oregon Health and Science University (OHSU), Portland, Oregon, USA.; 3 Department of Pathology, Stanford University School of Medicine, Stanford, California, USA.; 4 Department of Research Pathology, Genentech, South San Francisco, California, USA.; 5 Vanderbilt-Ingram Cancer Center and; 6 Department of Pathology, Microbiology and Immunology, Vanderbilt University Medical Center (VUMC), Nashville, Tennessee, USA.; 7 Department of Biochemistry, Vanderbilt University, Nashville, Tennessee, USA.; 8 Cancer Early Detection Advanced Research Center, OHSU, Portland, Oregon, USA.

**Keywords:** Immunology, Oncology, Breast cancer, Macrophages, T cells

## Abstract

Spatial profiling of tissues promises to elucidate tumor-microenvironment interactions and generate prognostic and predictive biomarkers. We analyzed single-cell spatial data from 3 multiplex imaging technologies: cyclic immunofluorescence (CycIF) data we generated from 102 patients with breast cancer with clinical follow-up as well as publicly available mass cytometry and multiplex ion-beam imaging datasets. Similar single-cell phenotyping results across imaging platforms enabled combined analysis of epithelial phenotypes to delineate prognostic subtypes among patients who are estrogen-receptor^+^ (ER^+^). We utilized discovery and validation cohorts to identify biomarkers with prognostic value. Increased lymphocyte infiltration was independently associated with longer survival in triple-negative (TN) and high-proliferation ER^+^ breast tumors. An assessment of 10 spatial analysis methods revealed robust spatial biomarkers. In ER^+^ disease, quiescent stromal cells close to tumor were abundant in tumors with good prognoses, while tumor cell neighborhoods containing mixed fibroblast phenotypes were enriched in poor-prognosis tumors. In TN disease, macrophage/tumor and B/T lymphocyte neighbors were enriched, and lymphocytes were dispersed in good-prognosis tumors, while tumor cell neighborhoods containing vimentin^+^ fibroblasts were enriched in poor-prognosis tumors. In conclusion, we generated comparable single-cell spatial proteomic data from several clinical cohorts to enable prognostic spatial biomarker identification and validation.

## Abstract 2

The developed methods enable assembly of larger clinical cohorts from diverse high-plex imaging platforms to aid in prognostic spatial biomarker identification and validation.

## Graphical abstract

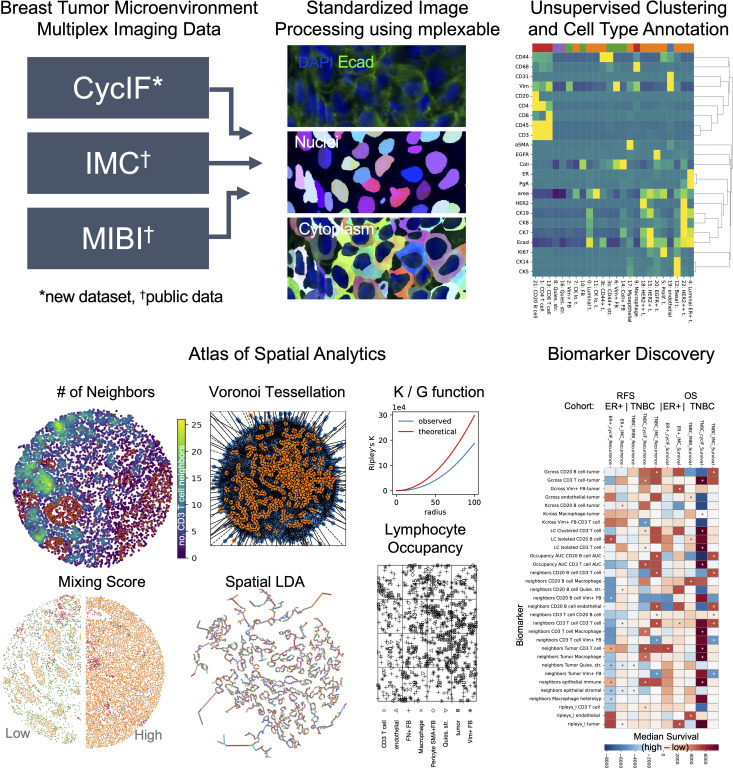

## Introduction

Recent advances in the breast cancer treatment landscape have motivated the characterization of the breast tumor microenvironment for deeper understanding of tumor-stroma interactions. For example, identifying biomarkers predicting breast cancer immunotherapy response is still an urgent clinical need (1). In metastatic TNBC, only a quarter of PD-L1^+^ patients respond to single-agent immune checkpoint blockade (1). In contrast, in early-stage TNBC, response rates to neoadjuvant immune checkpoint plus chemotherapy were similar in PD-L1^+^ and PD-L1^–^ groups (2). This highlights the need for biomarker development for better patient stratification across disease stages and spatial characterization for improved understanding of tumor-microenvironment interactions in resistant patients.

Highly multiplex imaging methods enable quantification of dozens of biomarkers in a single tissue section at subcellular resolution while retaining spatial context (3–8). Tissue structures such as tertiary lymphoid structures, identified with multiplex imaging, are predictive biomarkers of immunotherapy response in melanoma (9, 10). Spatial proximity between tumor, immune, and stromal cell types is associated with response to neoadjuvant therapy in human epidermal growth factor receptor 2^+^ (HER2^+^) breast cancer (11). In several breast cancer multiplex imaging studies, single-cell spatial context has prognostic relevance and shows correlations with transcriptomic and genomic features of tumors (12–15).

However, spatial biomarkers can be difficult to reproduce due to limited numbers of patients used to develop them and difficulties in comparing data from different imaging platforms. Furthermore, overfitting is an issue in biomedical imaging data due to the number of steps in the processing pipeline and the number of variables and parameters involved. Overfitting can be addressed through use of a discovery cohort to tune analytical methods, which are then fixed and subsequently applied to a validation cohort (16). In theory, validation cohorts can be readily obtained by incorporating publicly available data from disparate imaging platforms into biomarker studies. In practice, integrated analysis of such data remains a challenge. Furthermore, metadata documentation, analysis protocols, and code are essential for data reuse and reproducibility of findings, preferably using open-source software tools (16). We developed an open-source python software, mplexable (17), for multiplex image processing and analysis, which we use herein to process and analyze 3 multiplex imaging breast cancer cohorts: a cyclic immunofluorescence (CycIF) dataset that we generated and publicly available imaging mass cytometry (IMC) and multiplex ion-beam imaging (MIBI) datasets (12, 14, 18). This proof-of-concept study identifies prognostic single-cell spatial biomarkers common across imaging platforms. As imaging datasets become more widely available, our tools can facilitate biomarker discovery with high accuracy, reliability, and efficiency.

## Results

### Multiplex imaging datasets from different platforms produce similar single-cell phenotypes.

**Figure 1:**
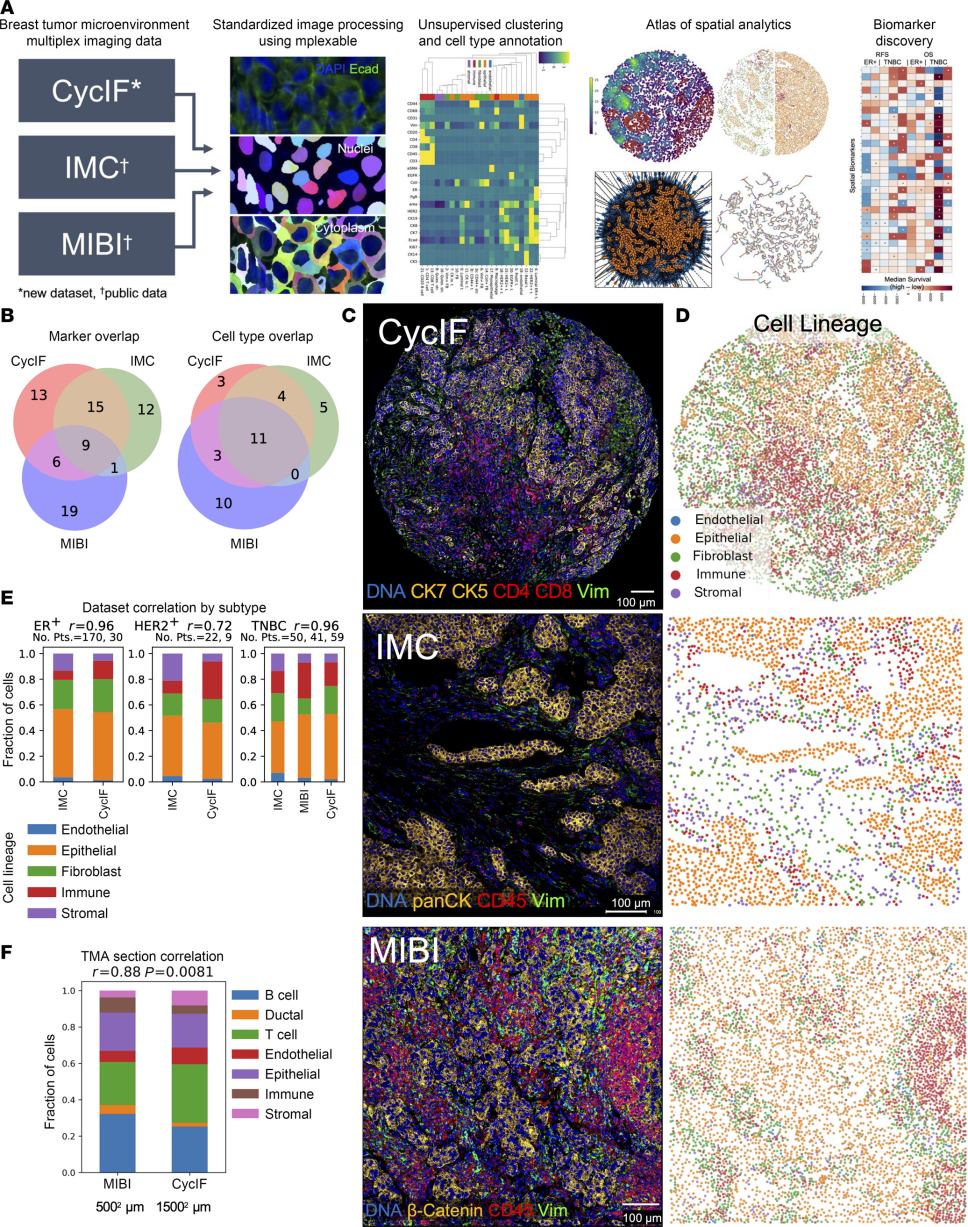
Concordant cell phenotypes in multiplex imaging data from different platforms. (**A**) Three multiplex imaging datasets from breast cancer tissue microarrays were processed through single-cell segmentation and feature extraction using the mplexable pipeline. The single-cell datasets were separately clustered using the unsupervised Leiden algorithm resulting in cell types that were annotated with similar names across platforms. We generated a suite of spatial statistics for each tissue, and the resulting cellular and spatial features were used for discovery and validation of prognostic cell abundance and spatial biomarkers across datasets. CycIF, cyclic immunofluorescence; IMC, imaging mass cytometry; MIBI, multiplex ion beam imaging. Asterisk indicates a new dataset, and cross indicates public data. (**B**) Overlap of markers (left) and annotated cell types (right) in each multiplex imaging dataset. (**C**) Representative images from the 3 multiplex imaging platforms showing epithelial (orange), immune (red), and fibroblast (green) markers. Scale bar: 100 μm. Total of 413 patient tissues imaged. (**D**) Cell lineage types showing cell location and lineages: epithelial (orange), immune (red), fibroblast (green), endothelial (blue), and other stromal (purple). CycIF, top; IMC, middle; MIBI, bottom. (**E**) The correlation between platforms of the fraction of each cell lineage per total cells per subtype, per platform, using unsupervised clustering and annotation to determine lineage. No. Pts., number of patients. (**F**) Correlation between cell types on adjacent sections of a TMA stained with MIBI and CycIF. *n* = 9 tissues. (**E** and **F**) Pearson’s correlation *r* (2-sided) between platforms and *P* value shown in panel title.

We generated CycIF data from 2 tissue microarrays (TMAs) containing surgical breast cancer specimens and obtained publicly available breast cancer multiplex images generated with antibody panels targeting similar antigens from IMC and MIBI platforms (Figure 1, A and B). Image processing was standardized across all 3 datasets using our mplexable pipeline (Figure 1A). The CycIF data were composed of 42 biomarkers imaged at a resolution of 0.325 μm per pixel in entire cores with 1.2–1.4 mm diameters, with 1–2 full cores imaged per patient (Figure 1C). The IMC data include 35 biomarkers imaged in the largest square area contained within the TMA cores (0.6–0.8 mm diameter), at a resolution of 1 μm per pixel (Figure 1C) (14). The MIBI data include 36 biomarkers imaged in 0.8 × 0.8 mm square regions of interest (ROIs) at a resolution of 0.5 μm per pixel (Figure 1C) (12).

Clinicopathological information was available for the IMC and CycIF datasets. Stage, tumor size, and patient age at diagnosis were available for 364 patients in the CycIF and IMC datasets, and grade was available for 275 patients in the IMC dataset (Supplemental Figure 1A; supplemental material available online with this article; https://doi.org/10.1172/jci.insight.176749DS1). Similar fractions of early- and advanced-stage patients were analyzed by IMC and CycIF, apart from the CycIF triple-negative breast cancer (TNBC) group lacking patients at stage 4 (Supplemental Figure 1B). Treatment information was available for the CycIF dataset; 16 patients with TNBC and 1 patient who is ER^+^ were treated with neoadjuvant chemotherapy (NAC). Patients who were treated with NAC had shorter recurrence-free survival (RFS) with no difference in overall survival (OS) (Supplemental Figure 1C). Multivariable Cox proportional hazards (CPH) modeling revealed that tumor size but not NAC independently predicted RFS (Supplemental Figure 1C), and patients treated with NAC had larger tumors (*P* < 0.001, Supplemental Figure 1D). Consistent with this, NAC-treated tissues had a higher fraction of Ki67^+^ proliferating cells within the epithelial compartment as well as a lower fraction of vimentin^+^ (Vim^+^) fibroblasts in the nonepithelial stroma; however, these were not significant after correcting for multiple testing (Supplemental Figure 1E). These results are consistent with NAC being given to downsize tumors before surgery in a subset of patients.

We used the same analytical methods for each platform to generate single-cell phenotypes via unsupervised Leiden clustering (19) followed by visual inspection and annotation. We validated this method on serial breast cancer TMA slides approximately 20 mm apart stained with similar CycIF panels on different dates (17). Across 5 cores, the average Pearson correlation *r* value between cell type fractions was 0.99, with a SD of 0.02, demonstrating excellent reproducibility (Supplemental Figure 1, F and G).

In the CycIF dataset, we clustered cells using 23 markers plus nuclear area. We visualized the single cells using a uniform manifold approximation and projection (UMAP) embedding, confirming good separation of lineage specific markers: CD31, endothelial; E-cadherin (Ecad), epithelial; Collagen I (ColI), extracellular matrix; Vim, mesenchymal cells including activated fibroblasts; and CD45, immune infiltrate (Supplemental Figure 2A). The UMAP visualization shows good mixing of cells from different TMA sources, indicating minimal batch effects, and separation of tumor cells from different breast cancer subtypes (Supplemental Figure 2B). Clustering resulted in 23 cell type clusters (Supplemental Figure 2C). The mean expression of each biomarker in each cluster was used to annotate cell types and lineages (i.e., endothelial, epithelial, fibroblast, immune, and other stromal; Supplemental Figure 2D). The most common cell types included luminal and luminal ER^+^ tumor, CD4 T cells, Vim^+^ fibroblasts, and quiescent stroma (Supplemental Figure 2D). To confirm our clustering-based cell typing, we performed manual gating on lineage-specific markers (Supplemental Figure 2E). Gating and clustering-based cell lineages localized to similar areas of the UMAP and had 73% agreement on a single-cell level, as calculated using metrics.accuracy_score in scikit-learn (20) (Supplemental Figure 2, E and F).

As in the CycIF dataset, we visualized lineage marker expression, TMA batch effects, breast cancer subtype, and clustering results on a UMAP in the IMC dataset, confirming separation of cell lineages and mixing of cells from separate TMAs (Supplemental Figure 3, A–C). We used 21 markers plus nuclear area for clustering, resulting in 24 cell types (Supplemental Figure 3D). Upon annotation, we found the most common cell types were similar to those in the CycIF samples, namely luminal, luminal ER^+^ and ER^+^HER2^+^ tumor, Vim^+^ or fibronectin^+^ (FN^+^) fibroblasts, quiescent stroma, and T cells (Supplemental Figure 3D). Clustering and gating-based cell lineages localized similarly on a UMAP and had 77% agreement (Supplemental Figure 3, E and F).

The MIBI panel included more immune-specific markers than the other panels and had only 15 markers shared with our CycIF panel (Figure 1B). To audit deeper immune contexture, we clustered using all 33 markers plus nuclear area and eccentricity. Again, we visualized lineage-specific markers and clustering with a UMAP to confirm separation of cell lineages and annotated 22 cell types of which luminal tumor, fibroblasts, T cells, and quiescent stroma were the most common, similar to CycIF and IMC (Supplemental Figure 4, A–C). Gating and clustering-based localized similarly on a UMAP and had 72% agreement (Supplemental Figure 4, D and E).

### Cell-type fractions are similar across platforms.

While the most common cell types were similar across platforms, for further validation, we compared the fractions of the 5 main cell lineages across platforms. In all 3 platforms, cell lineage identity and spatial distribution matched the underlying imaging data (Figure 1, C and D). We calculated the fraction of cells in each lineage for each platform and breast cancer subtype. Both gating and clustering cell types showed high correlation (Pearson *r* = 0.97 gating and 0.96 clustering) across platforms for ER^+^ (*n* = 30 CycIF, 170 IMC) and TNBC (*n* = 59 CycIF, 50 IMC, 41 MIBI), while HER2^+^, which had a smaller number of samples (*n* = 8 CycIF, 22 IMC) had more variability between platforms (Figure 1E and Supplemental Figure 5A). We did note some platform-specific bias; for example, IMC showed a smaller fraction of immune cells defined by clustering in all 3 subtypes (Figure 1E). Therefore, when setting high/low cutoffs for cell abundances, we calculated high and low relative to each platform and subtype, as opposed to the whole dataset. Since different antibody clones, probes, and imaging systems were used, resulting in different signal/background ratios between platforms, even for the same target, we believe this is necessary to account for technical variability.

As an additional validation, we obtained CycIF and MIBI data collected from sections of the same TMA containing normal tonsil, lymph node, liver, pancreas, and placenta tissues. CycIF data were collected from the entire TMA core, while MIBI data were collected from 500 × 500 mm ROIs in each core (Supplemental Figure 5B). Despite the order of magnitude difference in the number of cells analyzed, the MIBI and CycIF data show good concordance, with an overall Pearson correlation of 0.88 (*P* = 0.008; Figure 1F). Cell types showed similar spatial localization between platforms and general consistency across the 9 cores analyzed (mean Pearson’s *r* = 0.89, SD = 0.13; Supplemental Figure 5, B and C).

Finally, we considered whether heterogeneity across the tumor would confound observations of cell type abundances from TMA cores. We collected CycIF data from full tissue sections of an ER^+^ and a TNBC tumor (21), and we selected ROIs from within the tumor core, on the tumor/stroma border, and in the adjacent normal tissue (Supplemental Figure 5, D and E). As expected, we found significant differences in epithelial and stromal cell phenotypes between tumor and adjacent normal tissue after normalizing for epithelial content (Supplemental Figure 5F). In contrast, in both tumor sections, we found no significant difference in cell type abundance in the tumor core and at the tumor/stroma border after normalizing for the number of epithelial cells (Supplemental Figure 5, G and H). Without normalizing for epithelial content, border ROIs in TNBC had more CD8 T cells and quiescent stroma (FDR < 0.05) and trended toward more B cells and macrophages (FDR < 0.06), while tumor ROIs had more luminal tumor (Supplemental Figure 5I). Intratumoral heterogeneity is a concern when analyzing TMAs, but if we normalize for epithelial content, variations in the location of the TMA core punch — i.e., closer to the tumor core or the tumor/stroma border — had no substantial effect on cell type abundance in this small sample.

### Unsupervised clustering defines prognostic tumor subtypes consistent across platforms.

**Figure 2:**
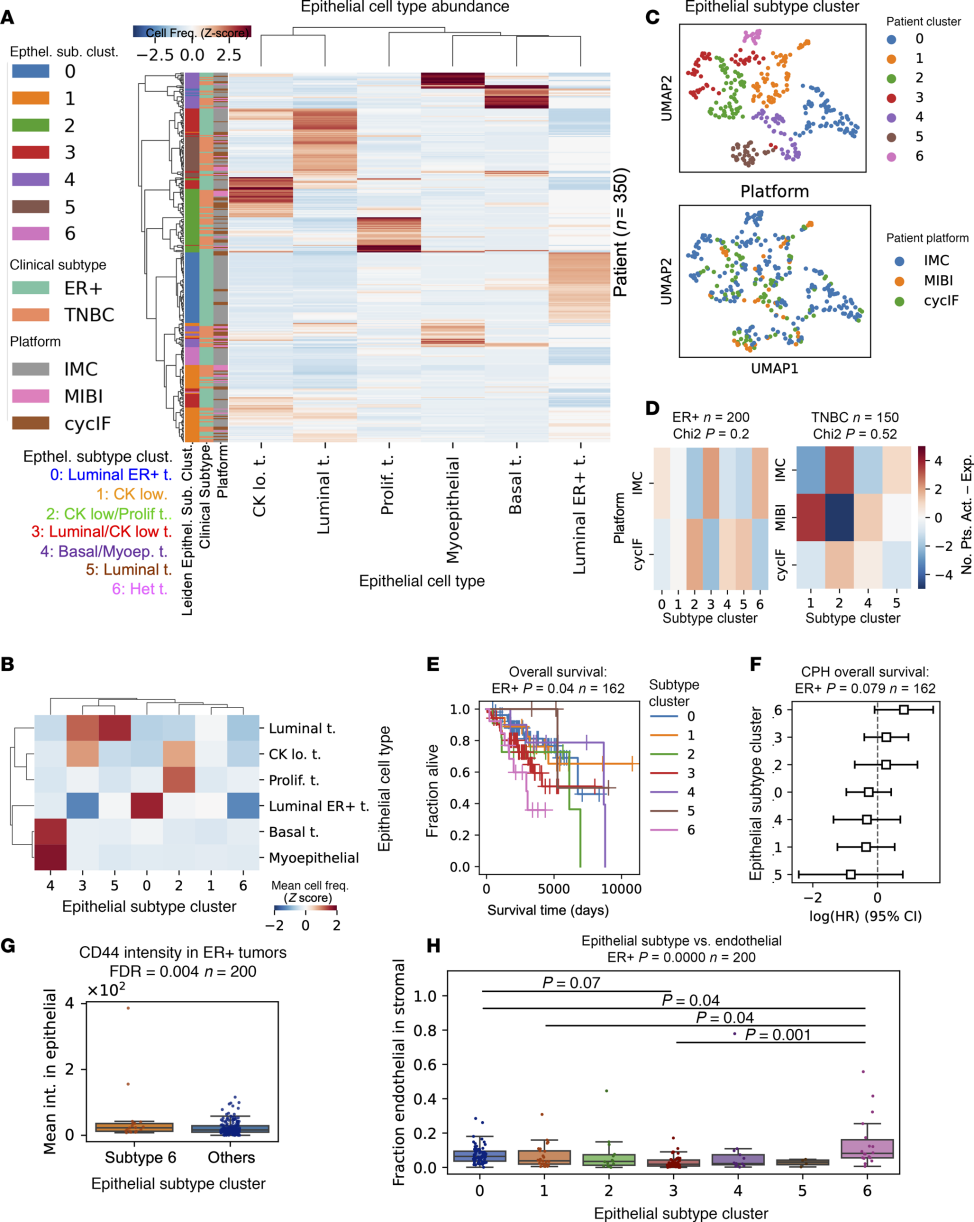
Prognostic ER^+^ breast cancer subtypes in multiplatform multiplex imaging data. (**A**) Hierarchical clustering of all ER^+^ and patients with TNBC (*n* = 350) based on the *Z*-scored fraction in each patient’s tissue of the 6 most common epithelial cell types. Heatmap annotation row colors show the Leiden clustering resulting in 7 epithelial (Epithel.) subtype clusters (left), clinical subtype (center), and platform (right). (**B**) Mean cell frequency of epithelial cell types per subtype cluster. (**C**) UMAP embedding of patients by fraction of epithelial cell types in all tumor cells, colored by Leiden epithelial subtype cluster (top) and platform (bottom). *n* = 350 patients. (**D**) Two-sided χ^2^ analysis of epithelial subtypes versus platform; *P* values are shown in panel title. (**E**) Kaplan-Meier curves (*P* value from log-rank test) comparing overall survival (OS) in the 7 epithelial subtypes present in ER^+^ tumors. (**F**) Cox proportional hazard (CPH) model estimating HRs for epithelial subtypes of ER^+^ tumors. The HR estimates marked by boxes and data are shown with 95% CI. (**G**) CD44 intensity in epithelial cells from poor prognosis epithelial subtype 6 compared with other patients who are ER^+^. FDR corrected for multiple cell markers given in panel title; *P* values were calculated from Mann-Whitney *U* test. (**H**) Fraction of endothelial cells in tissue stromal cells of patient tissues from each epithelial subtype cluster. Kruskal-Wallis *P* value is given in panel title. Post hoc Tukey’s HSD *P* values for pairwise comparisons were used between groups. (**G** and **H**) Box plots show the median and interquartile range (IQR), and whiskers show 1.5× the IQR.

First, we examined the prognostic value of epithelial cell type–based subtypes. To normalize for epithelial content, we calculated the fraction of each epithelial cell phenotype in the total epithelial cells in that sample. We then normalized across platforms by *Z*-scoring cell fractions within each platform (Supplemental Figure 6, A–D). For subtyping based on single-cell epithelial phenotypes, *Z*-scored fractions of epithelial cell types (excluding rare cell types < 4% of total) were used to Leiden cluster patients who are ER^+^ and patients with TNBC from all platforms (Figure 2A and Supplemental Figure 6E). The resulting 7 clusters included tissues enriched for luminal, basal, luminal ER^+^, myoepithelial, cytokeratin-low, and proliferating tumor cells, as well as a heterogeneous group not dominated by 1 phenotype (Figure 2, A and B, and Supplemental Figure 6F). Each subtype cluster contained a mixture of patients from multiple platforms, with no significant relationship between platform and subtype (χ^2^, *P* = 0.2 ER^+^, *P* = 0.52 TNBC; Figure 2, C and D). The epithelial subtypes present in the patients who are ER^+^ were prognostic (log-rank OS, *P* = 0.04, *n* = 162 patients; Figure 2E). CPH modeling showed that the heterogeneous subtype 6 had a hazard ratio (HR) > 1, suggesting poor prognosis (*P* = 0.079; Figure 2F). The CPH HRs were similarly ordered across the IMC and CycIF cohorts, with heterogeneous subtype 6 having a HR > 1 in both platforms (Supplemental Figure 7, A and B). Investigation of the poor prognosis subtype 6 tissues revealed expression of CD44 and EGFR in ER^+^ tumor cells (Supplemental Figure 7C). Quantification of epithelial marker expression showed increased CD44 and EGFR expression in subtype 6 but not more epithelial heterogeneity quantified by Shannon entropy (FDR < 0.05; Figure 2G and Supplemental Figure 7, D and E). We asked whether poor prognosis subtype 6 harbored any distinctive stromal cell types. Quantification showed significant enrichment of CD31^+^ endothelial cells in subtype 6 (Tukey’s HSD, *P* < 0.05; Figure 2H). In patients with TNBC, the epithelial subtypes were not significantly associated with prognosis (Supplemental Figure 7, F–H).

### Microenvironment subtypes associate with clinical subtypes.

Next, we examined the prognostic value of stromal cell type–based subtypes. We clustered patients based on the stromal cell type fraction in each tissue in a similar manner to the epithelial subtyping above, selecting *k* = 6 for the number of clusters (Supplemental Figure 8, A–C). Since the stromal phenotypes differed across platforms due to different markers, we clustered patients from each platform separately, using the fractions of stromal cell types representing greater than 2% of the stromal compartment. The stromal subtypes were not prognostic, except for the MIBI platform (log-rank = 0.003; CPH, *P* = 0.056; *n* = 39 patients; Supplemental Figure 8, D–F). However, we observed significant correlation between stromal subtypes and clinical subtypes ER^+^ and TNBC. In the CycIF cohort, patients who are ER^+^ had significantly more of the Vim^+^ stromal subtype 0 and less T cell–rich stroma compared with TN (χ^2^, *P* = 0.098; Bonferroni, *P*_adj._ < 0.05 for subtype 0; *n* = 89 patients; Supplemental Figure 8G). Similarly, in the IMC cohort, patients who are ER^+^ had more Vim^+^/FN^+^ fibroblast stromal subtype 0 and significantly less T cell stromal subtype 4 compared with TN (χ^2^, *P* = 0.002, *P*_adj._ < 0.05 for subtype 4; *n* = 220 patients; Supplemental Figure 8H). Our characterization of stromal subtypes supports the observation that ER^+^ breast cancer is immune-poor (22) and shows significant enrichment for Vim^+^ and FN^+^ fibroblasts relative to TNBC.

### T cells are an independent prognostic factor in TNBC and high-proliferation ER^+^ tumors.

**Figure 3:**
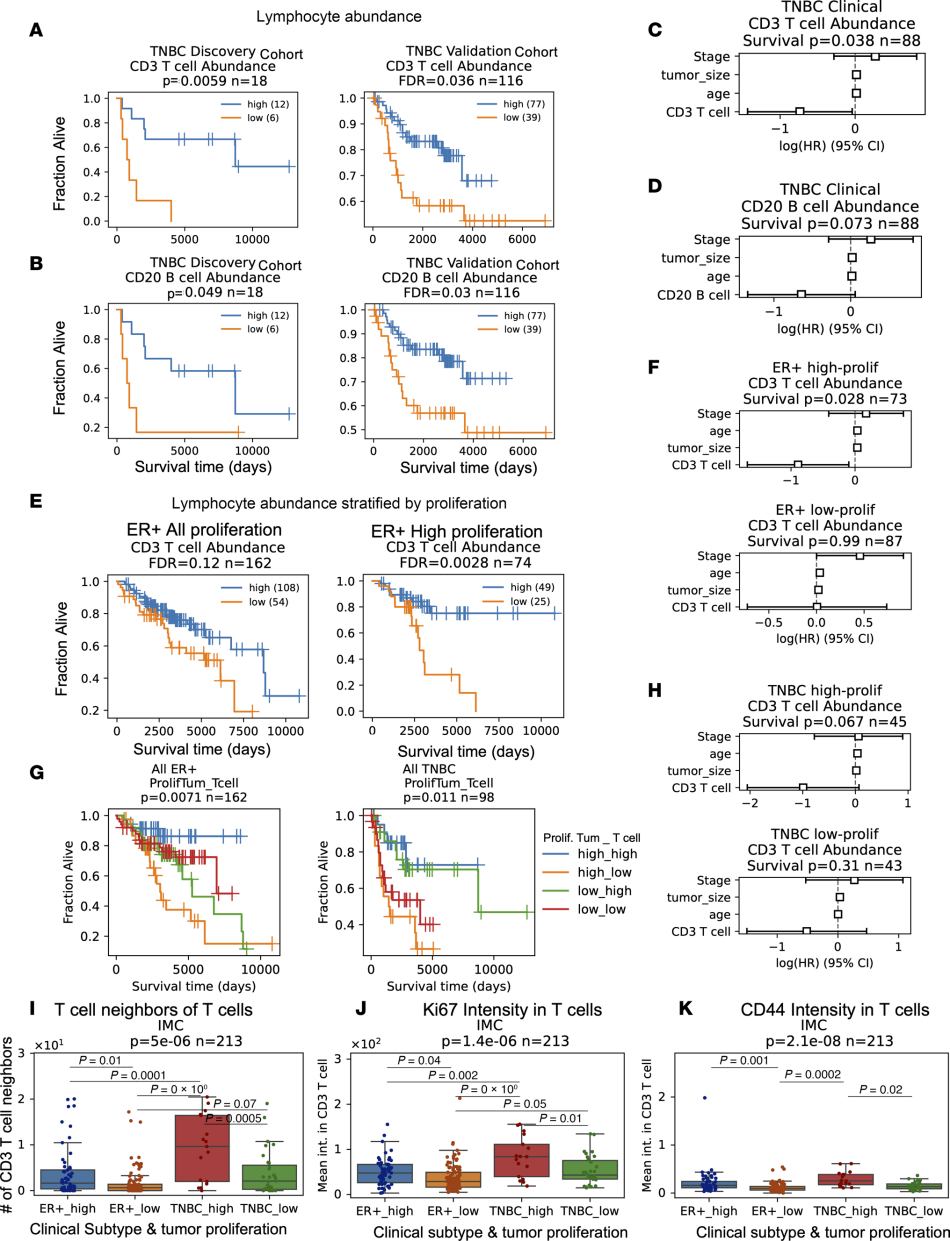
T cell infiltrate has prognostic value and distinct states in TN and high-proliferation ER^+^ breast cancer. (**A**) Kaplan-Meier analysis of abundance of CD3 T cells versus overall survival (OS) in TNBC discovery (left) and validation cohort (right). (**B**) Kaplan-Meier analysis of abundance of CD20 B cells versus OS in TNBC discovery (left) and validation cohort (right). (**C**) Multivariable CPH modeling adding patient age, tumor size, and stage to CD3 T cell high variable defined in **A**. (**D**) Multivariable CPH modeling adding patient age, tumor size and stage to CD20 B cell high variable defined in **B**. (**E**) Kaplan-Meier analysis of abundance of CD3 T cell versus OS in all patients who are ER^+^ (left) and patients who are ER^+^ with high (above the median) tumor proliferation (right). (**F**) CPH modeling of CD3 T cell abundance plus clinical variables in high- and low-proliferation ER^+^ tumors. (**G**) Kaplan-Meier analysis of all patients who are ER^+^ and patients with TNBC stratified into 4 groups by median tumor proliferation and median T cell abundance. (**H**) CPH modeling of CD3 T cell abundance plus clinical variables in high- and low-proliferation TNBC tumors. (**I**) Mean number of T cell neighbors (within 25 μm) of T cells in tissues from high- and low-proliferation ER^+^ or TNBC tumors in IMC cohort. (**J**) Ki67 intensity indicating proliferation levels of T cells in tissues from high- and low-proliferation ER^+^ or TNBC tumors in IMC cohort. (**K**) CD44 intensity in T cells, indicating memory/effector phenotypes in IMC tissues. (**A**–**H**) All Kaplan-Meier *P* values obtained from the log-rank test, validation cohort corrected for testing multiple cell types with Benjamini-Hochberg method. CPH modeling *P* values for cell type variable given in panel titles; the HR estimates are marked by boxes, and data are shown as 95% CI. (**I**–**K**) Significance was found with the Kruskal-Wallis test. Post hoc Tukey’s HSD was used for pairwise comparisons between groups. Box plots show the median and interquartile range (IQR), and whiskers indicate 1.5× the IQR.

To further investigate the prognostic value of multiplex imaging–defined cell types, we utilized a discovery cohort to tune analytical methods, which were then fixed and subsequently applied to a validation cohort (16). The CycIF TMA1 dataset served as a discovery cohort to identify cell types whose fraction in the tissue were significantly associated with OS and determine cut-offs for prognostic high/low abundance. We tested the 0.33, 0.5, and 0.66 quantile to binarize tissues into low and high cell abundance (Supplemental Figure 9). For any cell type showing prognostic significance (log-rank, *P* < 0.05), we selected the cut-off with the lowest *P* value for validation in the other datasets, correcting for testing of multiple cell types using the Benjamini-Hochberg method. Using this methodology, we found that a high abundance of T and B cells were associated with longer OS in TNBC in both the discovery and validation cohorts (validation FDR < 0.05; Figure 3, A and B).

Prognostic biomarkers significant as a single variable (validation log-rank FDR < 0.05) were combined with the clinical variables of stage, patient age, and tumor size in a multivariable CPH model. In TNBC samples with clinical data, high CD3 T abundance remained significantly associated with longer OS in the multivariable model (CPH, *P* = 0.038; *n* = 88; Figure 3C). High CD20 B cell abundance trended toward longer OS in the multivariable model (CPH, *P* = 0.073; *n* = 88; Figure 3D).

CD3 T cells were not associated with OS in the patients who are ER^+^ with breast cancer (FDR = 0.12, *n* = 162); however, in tumors with proliferation above the median, increased CD3 T cells were associated with longer OS (FDR = 0.0028, *n* = 74) (Figure 3E). Multivariable CPH modeling revealed that high CD3 T cells were independently prognostic for longer OS in high-proliferation but not low-proliferation ER^+^ tumors (CPH, *P* = 0.028 high proliferation, *P* = 0.99 low proliferation; Figure 3F). Separation of tumors into high and low by median proliferation and median T cell abundance showed that, in ER^+^ disease, high proliferation, high T cell tumors had the best prognosis, while high proliferation, low T cell tumors had the worse prognosis (log-rank, *P* = 0.0071; Figure 3G). In TNBC, both tumors with high proliferation and low proliferation (i.e., above and below the median, respectively), had similarly good survival if they had high T cells (i.e., above the median), while low–T cell tumors had similarly poor survival regardless of proliferation status (log-rank, *P* = 0.011; Figure 3G). Multivariable CPH modeling revealed that patients with high CD3 T cell abundance trended toward longer OS for high-proliferation but not low-proliferation TNBC tumors (CPH, *P* = 0.067 and 0.31; Figure 3H). Survival analysis in each platform revealed outcome stratification by T cell abundance and proliferation in patients who are ER^+^ from the IMC cohort but not the CycIF cohort (log-rank, *P* = 0.028 and 0.4), and in patients with TNBC from the IMC and CycIF cohorts but not MIBI cohort (log-rank, *P* = 0.056, 0.012, and 0.49; Supplemental Figure 10, A and B).

### T cells in patients deriving a survival benefit from infiltration have distinct functional states.

To elucidate T cell functional states present in patients deriving a survival benefit from T cell infiltration, we compared T cell spatial localization and marker expression in T cell–infiltrated groups with different survival outcomes. There was no significant difference in T cell abundance or T cell/macrophage or T cell/endothelial ratio in high- versus low-proliferation ER^+^ tumors (Supplemental Figure 10C). Interestingly, high-proliferation ER^+^ tumors in the IMC cohort, which gained survival benefit from CD3 T cells, showed more clustering of CD3 T cells than low-proliferation ER^+^ tumors, quantified by the mean number of T cell neighbors of each T cell (ER^+^ high versus low proliferation Tukey’s HSD, *P* = 0.01; Figure 3I). High-proliferation ER^+^ tumors in the CycIF cohort, which did not gain a survival benefit from CD3 T cells, did not show increased T cell clustering (Kruskal-Wallis, *P* = 0.61; Supplemental Figure 10D). Similarly, CD3 T cells in high-proliferation ER^+^ tumors from the IMC cohort had higher levels of the proliferation marker Ki67 and the memory/effectormarker CD44 than in low-proliferation ER^+^ tumors, indicating a more activated functional state (ER^+^ high versus low proliferation Tukey’s HSD, P = 0.04 and 0.001; Figure 3, J and K). In the CycIF cohort, reduced Ki67 and CD44 expression on T cells was observed between ER^+^ and TNBC subtypes, consistent with an activated T cell state correlating with a survival benefit derived from increased T cell infiltration (Supplemental Figure 10E). High-proliferation TNBC, in the MIBI cohort, showed increased levels of PD-1, FoxP3, IDO, and Lag3 expression in T cells, consistent with upregulation of negative feedback checkpoints following immune activation (Supplemental Figure 10, E and F). Epithelial cells in high-proliferation TNBC in the MIBI cohort had increased expression of the antigen presentation molecule HLA Class 1 and immune checkpoint PD-L1 (Supplemental Figure 10G).

### Analysis of tumor-stromal proximity reveals robust spatial biomarkers.

**Figure 4:**
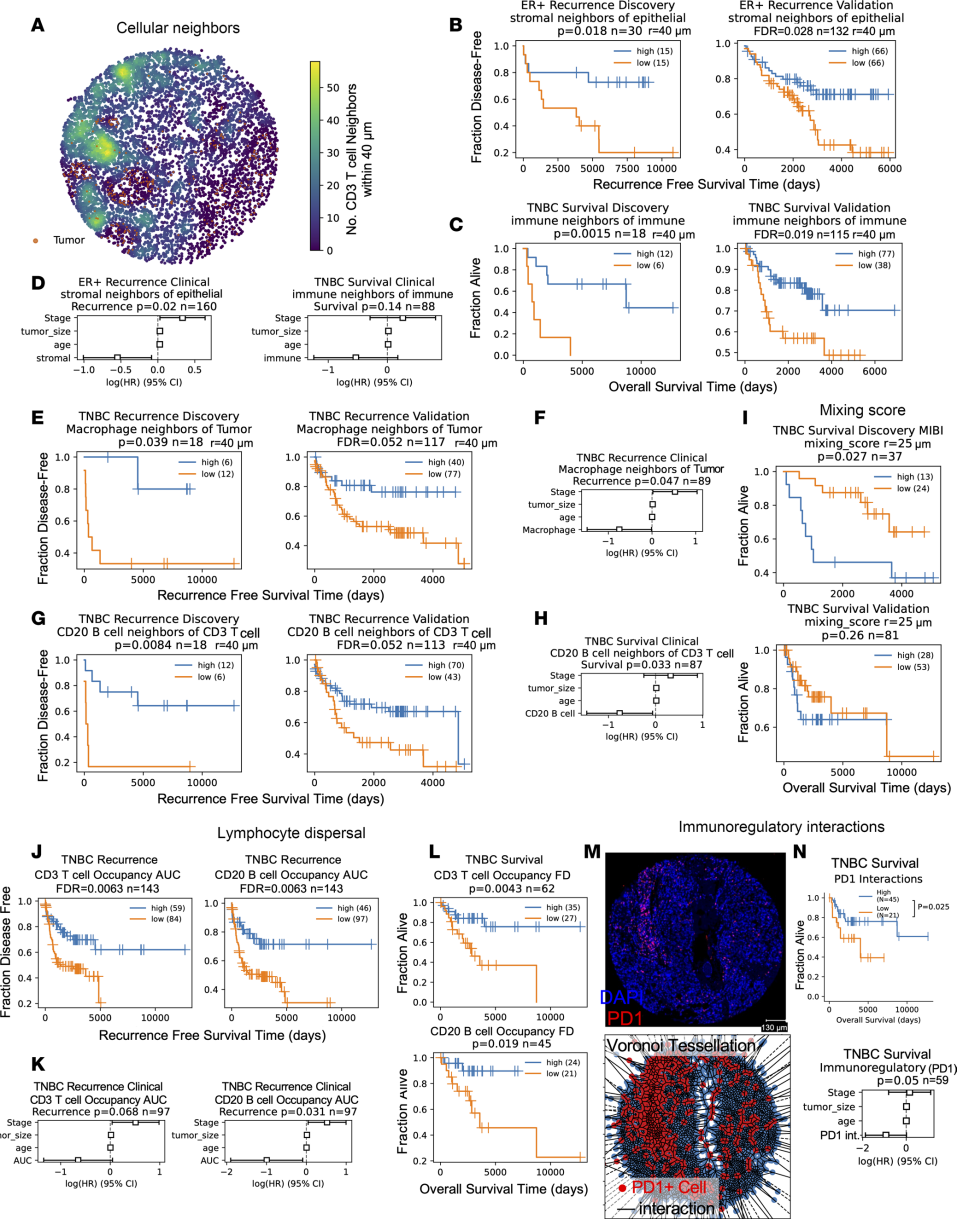
Reproducible prognostic spatial metrics in breast cancer cohorts. (**A**) Example tissue colored by number of CD3 T cell neighbors of each cell. Tumor in orange. (**B** and **C**) Recurrence-free survival (RFS) of patients who are ER^+^ stratified by stromal neighbors of epithelial (**B**) or overall survival (OS) of patients with TNBC stratified by immune neighbors of immune (**C**) in the discovery (left) and validation (right) cohorts. (**D**) Multivariable CPH modeling of (left) RFS of patients who are ER^+^ for stromal neighbors of epithelial or (right) OS of patients TNBC for immune neighbors of immune. (**E** and **G**) RFS of patients with TNBC stratified by macrophage neighbors of tumor (**E**) or B cell neighbors of T cells (**G**) in the discovery (left) and validation (right) cohorts. (**F** and **H**) Multivariable CPH modeling of RFS of patients with TNBC for macrophage neighbors of tumor (**F**) or CD20 B cell neighbors of CD3 T cells (**H**). (**I**) TNBC OS stratified by tumor-immune mixing score in MIBI (top) and validation cohorts (i.e., CycIF and IMC; bottom). (**I**) TNBC OS stratified by occupancy AUC of T (left) or B lymphocytes (right). (**K**) Multivariable CPH modeling of T (left) and B lymphocyte (right) occupancy AUC. (**L**) TNBC OS stratified by fractal dimension slope difference for T (top) or B lymphocytes (bottom). (**M**) Top: Representative tissue showing nuclei (blue) and PD-1 (red). Scale bar: 130 μm. Bottom: Voronoi tessellation of tissue; all cells (blue), PD-1^+^ cells (red), and interactions (black line). (**N**) OS of CycIF TNBC patients stratified by PD-1 interactions. Bottom: CPH modeling of PD-1 interaction metric. (**A**–**N**) Kaplan-Meier *P* values from log-rank test; validation cohort FDR corrected with the Benjamini-Hochberg method. CPH modeling *P* values for spatial variable given in panel titles; HR estimates are marked by boxes, and data are shown as 95% CI. (**A**–**H**) Neighbors are within a 40 μm radius. (**J**–**L**) Includes lymphocytes within 20 μm of tumor.

Intrigued by the finding that T cells in high-proliferation tumors had increased T cell neighbors and were associated with a survival benefit (Figure 3I), we leveraged our discovery and validation cohort analysis to systematically investigate cellular spatial relationships as prognostic biomarkers in breast cancer. We calculated the number of immune, stomal, and tumor neighbors within proximity of each other to derive previously described biomarkers including mean neighbor counts (11, 13), tumor-immune mixing score (12), immunoregulatory interactions (18), lymphocyte clustering, and lymphocyte occupancy (15). We also used common statistical methods for quantification of spatial correlation (Ripley’s L, Kcross [multitype K], and Gcross [multitype nearest neighbor distance] functions) (23). Analysis of proximity between cells of different lineages revealed that increased stromal (i.e., nonfibroblast, nonimmune, nonendothelial) neighbors within 40 mm of epithelial cells predicted longer RFS in the discovery (log-rank *P* = 0.018) and validation ER^+^ cohorts (FDR = 0.028) and independently predicted RFS in a multivariable CPH model with clinical covariates (CPH *P* = 0.02), while increased immune neighbors within 40 mm of immune cells associated with longer OS in both TNBC cohorts (validation FDR = 0.019) but not in the multivariable model (CPH *P* = 0.14, Figure 4, A–D). Analysis of cell type neighbors showed increased macrophage neighbors within 40 mm of a tumor, and increased B cell neighbors within 40 mm of T cells trended toward longer RFS in TNBC in both cohorts (validation FDR = 0.052) and remained significant the multivariable model (CPH, *P* = 0.047 Macrophage-tumor, *P* = 0.33 B cell–T cell; Figure 4, E–H). Additional spatial metrics, including Ripley’s L, Kcross, and Gcross, did not yield any significant biomarkers in the validation cohort (Supplemental Figure 11A).

Previously, Ali et al. (13) showed that heterotypic neighbors of myofibroblasts, fibroblasts, cytokeratin low tumor cells, and Vim^+^ Slug-macrophages were associated with poor outcome, and homotypic neighbors of fibroblasts and myofibroblasts were associate with good outcomes in all breast cancer subtypes. We tested the prognostic value of heterotypic and homotypic neighbors of fibroblast subsets, cytokeratin-low tumors, and macrophages but did not find the significant association with survival (log-rank, FDR > 0.3; Supplemental Figure 11, B and C). Previously, Keren et al. (12) showed that a high tumor-immune mixing score was associated with poor survival in TNBC. We were able to reproduce with our cell typing the prognostic value of the mixing score in the MIBI cohort, where it was developed (log-rank, *P* = 0.027; Figure 4I, top). However, in a validation cohort containing patients in CycIF and IMC cohorts, the mixing score was not prognostic (log-rank, *P* = 0.26; Figure 4I, bottom) nor was it independently prognostic in samples with clinical outcome (CPH, *P* = 0.4; Supplemental Figure 11D).

Wortman et al. developed metrics for lymphocyte isolation and spatial dispersion which were linked to longer RFS in TNBC (15). Like Wortman et al., we found that in our TNBC cores, the majority (57%) of lymphocytes near tumor cells (i.e., within 20 μm) were isolated, defined as fewer than 5 lymphocytes per 20 μm radius, but unlike their findings, isolated lymphocytes near tumor in our data were not significantly associated with RFS (Supplemental Figure 11E). OS was significant, with greater numbers of isolated B cells near tumor cells associated with longer survival (log-rank, FDR = 0.014), which trended in a model with clinical covariables (*P* = 0.058; Supplemental Figure 11F). For lymphocytes near tumor cells, we calculated occupancy AUC, or AUC of lymphocyte quadrant counts at different length scales, and fractal dimension (FD) difference, determined from slope of the log-log plot of the number of squares with at least 1 tumoral lymphocyte versus the inverse box size, as described (15). Higher values of both metrics are indicative of spatial dispersion while lower values are associated with clustering. Similar to Wortman et al. (15), higher tumoral B and T cell occupancy AUC was associated with longer RFS in TNBC, which remained prognostic for B cells in the multivariable model (Figure 4, J and K). While FD differences could only be calculated in tissues with lymphocytes present near tumor and intact tissue > 200 μm^2^ sampled, higher tumoral T and B cell FD differences were associated as single variables with longer OS (Figure 4L), and T cell FD trended in the multivariable model (*P* = 0.063; Supplemental Figure 11G).

Formerly, Patwa et al. demonstrated in the MIBI cohort that increased spatial interaction between cells expressing immunoregulatory proteins PD-1, PD-L1, IDO, and Lag3 were associated with longer RFS (18). We repeated this analysis using PD-1 in the CycIF TNBC dataset and found that high PD-1 interactions were associated with longer OS but not RFS (log-rank, *P* = 0.025 and 0.2), and this trended in a multivariable model of OS with clinical covariates (CPH, *P* = 0.05; Figure 4, M and N, and Supplemental Figure 11H). Similar to Patwa et al., we found no prognostic value of lineage marker interactions (Supplemental Figure 11I). We were not able to replicate the prognostic value of functional protein interactions (log-rank OS, *P* = 0.06) and coexpression (log-rank OS, *P* = 0.38) that Patwa et al., previously reported (18) (Supplemental Figure 12, A and B), although the CycIF panel’s functional proteins only partially overlapped (7 of 18) with those analyzed in the MIBI dataset. Overall, our broad evaluation of spatial metrics across datasets revealed both examples of biomarkers with opposite survival associations in different platforms (Supplemental Figure 11A) and concordance with some previously identified biomarkers (15, 18), demonstrating the importance of a validation cohort (Supplemental Figure 12C).

To identify spatial metrics that provided additional information beyond abundance, we calculated the Pearson correlation between each spatial metric and cell type abundance within each patient’s tissue (Supplemental Figure 13). Stromal neighbors of epithelial (good prognosis in ER^+^) correlated with quiescent stroma abundance and macrophage neighbors of tumors (good prognosis in TNBC) correlated with macrophage abundance (Supplemental Figure 13). CD20 B cell neighbors of T cells, isolated lymphocytes and lymphocyte occupancy AUC (good prognosis in TNBC) correlated with each other and T and B cell abundance (Supplemental Figure 13). The tumor-immune mixing score was positively correlated with tumor abundance and negatively correlated with immune abundance (Supplemental Figure 13). Many of the Kcross and Ripley’s L function results correlated with each other and were not as strongly correlated with abundance (Supplemental Figure 13).

### Neighborhood analysis reveals multicellular spatial biomarkers.

**Figure 5:**
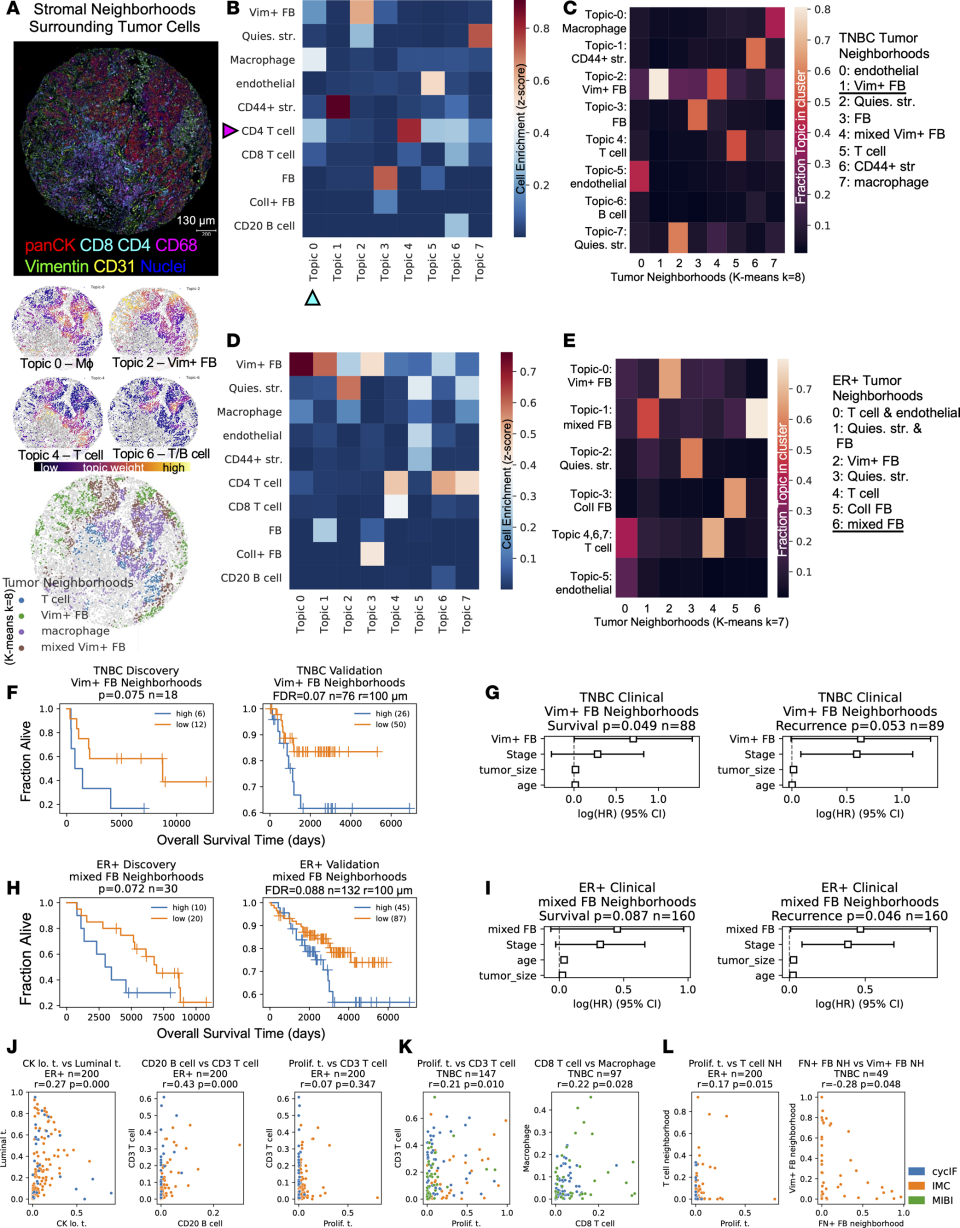
Prognostic multicellular neighborhoods surrounding tumor cells modeled with spatial latent Dirichlet allocation. (**A**) Top: CycIF staining of TNBC tissue showing tumor (panCK), T cell (CD4 and CD8), macrophage (CD68), fibroblast (vimentin), and endothelial (CD31) markers. Middle: Tumor cells colored by topic weights of select topics. Bottom: Tumor cells colored by their spatial latent Dirichlet allocation (LDA) neighborhood cluster. Tumor cells colored by the following neighborhoods: T cell (blue), macrophage (purple), mixed fibroblast (brown) and vimentin^+^ fibroblast (green). *n* = 308 patients analyzed with spatial LDA. (**B** and **D**) Heatmap of stromal cell enrichment in spatial LDA topics in a 100 μm radius of tumor cells in CycIF TNBC tissues (**B**) (*n* = 59) and ER^+^ tissues (**D**) (*n* = 30). Cyan arrowhead indicates cell type enrichment in topic-0 in TNBC tissues; magenta arrowhead indicates CD4 T cell enrichment in TNBC spatial LDA topics. (**C** and **E**) Heatmap of fraction of each topic in each neighborhood cluster resulting from K-means clustering (*k* =8) of spatial LDA topics from TNBC (**C**) and ER^+^ tissues (**E**). (**F**) Kaplan-Meier (K-M) estimate of overall survival (OS) for high and low vimentin^+^ fibroblast tumor neighborhoods in TNBC tissues in discovery (left) and validation cohorts (right). (**G**) CPH modeling of OS and recurrence-free survival (RFS) with clinical variables plus spatial LDA neighborhood from **F**. (**H**) K-M analysis of OS for high and low mixed fibroblast tumor neighborhoods in ER^+^ tissues in the discovery (left) and validation cohorts (right). (**I**) CPH modeling of OS and RFS for mixed fibroblast neighborhoods in ER^+^ tumors. (**J** and **K**) Pearson correlation of cell types within ER^+^ (**J**) and TNBC tissues (**K**) from all cohorts, colored by cohort (legend in **L**). Pearson correlation of neighborhood/cell type abundances, subtype in panel title, colored by cohort. (**F** and **H**) *P* values were calculated from the log-rank test. (**G** and **I**) CPH modeling *P* values for spatial variable are shown in panel titles; the HR estimates are marked by boxes, and data are shown as 95% CI. (**J**–**L**) Cell types, cohort, 2-sided Pearson correlation (*r*), and *P* values given in panel titles.

Finally, we analyzed multicellular spatial neighborhoods by considering stromal cells within a 100 mm radius of each tumor cell. We used spatial latent Dirichlet allocation (LDA) to model the neighborhood around each tumor cell as a combination of topics, utilizing a spatial parameter to increase the likelihood that adjacent cells share the same topics (24). LDA analysis can capture smoothly transitioning microenvironments (24) by assigning a probability for each topic to each neighborhood (Figure 5A). Each topic describes a microenvironment containing 1 or more cell type, and each cell type can be in 1 or more topic; for example, in CycIF data, topic-0 in TNBC tissues was enriched in macrophages, Vim^+^ fibroblasts, and CD4 T cells (Figure 5B, cyan arrowhead), while CD4 T cells are found in topic-0, -4, -5, and -6 (Figure 5B, magenta arrowhead). After topic modeling, K-means clustering was run on the single-cell topic matrix to define “tumor neighborhood” clusters that contained 1 or more topics (Figure 5, A–C). Clustering the topic matrix rather than the neighbor count matrix is believed to be less sensitive to noise (25). The spatial LDA neighborhood clusters were annotated based on their topics, and examination of the images showed that neighborhoods reflected the spatial distribution of the markers in the tissue (Figure 5A and Supplemental Figure 14, A–D). As expected, we observed transitioning/mixed neighborhoods within both TNBC and ER^+^ neighborhood cluster results (Figure 5, B–E). A TNBC tissue in our CycIF cohort, for example, showed tumor cell neighborhoods with more T cells on the tumor margin, with macrophage-rich neighborhoods in the tumor core (Figure 5A). These neighborhoods transition into a mixed neighborhood and finally a Vim^+^ FB neighborhood distant from the infiltrating T cells (Figure 5A). An ER^+^ tissue showed quiescent stroma neighborhoods in the tumor core, Vim^+^ FB neighborhoods on the tumor margin, and T cell neighborhoods in tumor nests isolated in the stroma (Supplemental Figure 14, A and D). Similar neighborhoods were identified in the IMC TNBC and ER^+^ cohorts (Supplemental Figure 14, E–H), but the MIBI cohort was excluded from neighborhood analysis due to lower stromal cell type overlap (Figure 1B). For survival analysis, we used the CycIF TMA1 as a discovery cohort and CycIF TMA2 plus IMC patients as a validation cohort. Due to the small sample size, we used an FDR threshold of 0.1 rather than 0.05 to report findings. Increased Vim^+^ FB neighborhoods around tumor cells were associated with shorter OS in both TNBC cohorts (validation log-rank FDR = 0.07; Figure 5F). Increased Vim^+^ fibroblast neighborhoods were associated with shorter OS and RFS in the multivariable model (CPH, *P* = 0.049 OS and 0.053 RFS; Figure 5G). Interestingly, Vim^+^ fibroblast abundance alone was not prognostic in TNBC (Supplemental Figure 14I). In ER^+^ tumors, increased mixed fibroblast neighborhoods containing VIM^+^ and VIM^–^ fibroblasts around tumor cells were associated with shorter OS (validation log-rank, FDR = 0.088) and trended significant in the multivariable model (CPH, *P* = 0.087 OS and 0.046 RFS; Figure 5, H and I). Finally, similar to other groups (25), we found that directly clustering the neighborhood counts using Kmeans (rather than running LDA and clustering the topics) did not result in robust prediction of prognosis (Supplemental Figure 15).

### Tumor phenotypes correlate with stromal cell abundance and spatial neighborhoods.

We hypothesized that there would be significant correlation between tumor cell types and the spatial LDA neighborhoods, correlations that could shed light on biologically and clinically relevant tumor-stroma crosstalk. First, we visualized a matrix of pairwise correlation between epithelial and stromal cell fractions and spatial LDA neighborhoods across subtypes (Supplemental Figure 16). Epithelial cell types were inversely correlated with each other (*P* < 0.05 TNBC, except basal-like; *P* < 0.001 ER^+^ disease), indicating most tumors had just one main epithelial cell type (Supplemental Figure 16). The exception was luminal tumor, which positively correlated with cytokeratin low tumor in ER^+^ breast cancer, indicating mixing of these ER^–^ phenotypes within the same tissues (*P* < 0.001; Figure 5J).

Immune cells exhibited distinct tissue-level correlations in the different subtypes. In ER^+^ breast cancer, T cells correlated with B cells (*P* < 0.001; Figure 5J), while proliferating tumor and macrophages correlated with endothelial cells (*P* < 0.01 and 0.05; Supplemental Figure 16B) but not T cells (*P* = 0.35; Figure 5J). In TNBC, T cells correlated with proliferating tumor (*P* = 0.01; Figure 5K), and macrophages correlated with CD8 T cells (*P* = 0.028; Figure 5K). Vim^+^, FN^+^, and ColI^+^ fibroblasts as well as quiescent stroma were inversely correlated with immune cells (*P* < 0.05; Supplemental Figure 16). In both subtypes, spatial LDA neighborhoods correlated strongly with the abundance of their respective stromal cell types; however, neighborhoods showed unique correlations to other cell types present. For example, proliferating tumor cell abundance did not correlate with T cell abundance in ER^+^ breast cancer, but it did correlate with the fraction of T cell neighborhoods (*P* = 0.015; Figure 5L). In TNBC, Vim^+^ and FN^+^ fibroblast abundance was not correlated (Supplemental Figure 16), but their respective neighborhoods were inversely correlated (*P* = 0.048; Figure 5L), suggesting exclusivity for a single fibroblast phenotype near tumor cells in each tissue. Therefore, although spatial neighborhoods tend to correlate with cell abundance, they can reveal unique features of tumor-stromal organization in tissues.

## Discussion

Our approach of standardized processing and analysis across multiple imaging platforms shows the power of our methods for biomarker discovery. We incorporated analysis of 2 publicly available imaging datasets with our own CycIF data for efficient discovery of robust biomarkers.

We utilized our validated method for CycIF staining and image processing (17) to generate multiplex imaging data of 42 markers in single tissue sections from 2 TMAs with clinical follow-up. Our dataset alone represents a valuable clinical cohort that provides improved plex, resolution, and ROI size compared with previously published datasets (12, 14). We then developed an analysis pipeline (https://github.com/engjen/cycIF_TMAs; commitID: 2bb6a6cbb8fd83bcbc87e900c0a0c823e1f1e51f) to generate single-cell phenotyping data from our CycIF dataset and 2 publicly available datasets (12, 14). The advantage of using our pipeline for image processing is the development of smoothing algorithms so that pixelated IMC and MIBI data can be segmented with deep-learning models trained on higher-resolution images, and an algorithm to match nuclear and cell segmentation results from separate deep-learning segmentation models to extract features from subcellular compartments such as the nucleus and cytoplasm. Using our methods, we generated single-cell data that produced a high correlation between cell types across cohorts from the same breast cancer subtype and also confirmed cell-type correlation on serial slides profiled with different platforms (Figure 1, E and F).

Additionally, we identified similar epithelial phenotypes across platforms and clustered patient data from all platforms to separate 7 epithelial subtypes without platform-specific bias (Figure 2). Our subtypes were consistent with the intrinsic breast cancer subtypes (26), including a luminal ER^+^ luminal A-like group with good prognosis and a cytokeratin-low group previously shown to share features with luminal B tumors (13). TNBCs also fell into categories similar to those defined by gene expression profiling (27), including a highly proliferative, basal-like 1–like (BL1-like) group, a luminal androgen receptor–like (LAR-like) group with luminal epithelial phenotypes, and a group with a basal/myoepithelial phenotype reminiscent of the basal-like 2 (BL2) group. We also identified a heterogeneous subtype with low cytokeratin and elevated CD44 expression that may represent tumors with mesenchymal features. Jackson et al. identified a similar single-cell pathology cluster of hormone receptor–positive mixed tumors with poor prognosis (14). Our analysis showed that the ER^+^ tumors in the heterogeneous subtype had a poor prognosis and increased angiogenesis. An EMT program in breast cancer cells is linked to increased vascular endothelial growth factor A expression, increasing angiogenesis and the capacity for tumor initiation (28), a mechanism that could explain these correlated tumor and stromal phenotypes and their association with poor outcome.

Tumor-infiltrating lymphocytes have been linked to good prognosis in TNBC (29), and we confirmed that T and B cells are independently prognostic in TNBC in the multiplex imaging datasets analyzed herein. Previous gene expression profiling studies link productive antitumor immunity and tumor proliferation. Nagalla et al. found that immune signatures were prognostic solely in patients with breast cancer with the highest proliferation gene expression (30). Subsequently, the same group showed that immune gene signatures were prognostic in highly proliferative basal-like, HER2-enriched and luminal B subtypes but not those with low proliferation (31). Similarly, we have shown that CD3 T cells are independently prognostic specifically in high-proliferation ER^+^ and TNBC tumors.

Our analysis of immune functional states showed increased T cell proliferation, activation, checkpoint molecule expression, and epithelial antigen presentation in high-proliferation tumors, consistent with IFN-γ pathway activation. Consistent with our analysis, gene network analysis previously showed activation of TNF-α/IFN-γ signaling pathways in tumors with productive antitumor immunity and TGF-β, an immunosuppressive cytokine, in tumors with unproductive antitumor immunity (31). TGF-β also has antiproliferative effects and is associated with good outcomes in ER^+^ breast cancer cohorts (32), suggesting that it could mechanistically link lower proliferation rates with immunosuppression and represent a rational drug combination with immune checkpoint targeting (33).

Thomas et al. recently showed that immune gene signatures were prognostic exclusively in tumor-mutation burden–high (TMB-high) breast cancer tumors (34). Thirty-seven percent of basal-like tumors had high TMB, while only 11.5% of luminal A tumors did (34), explaining the poor immunogenicity of the latter subtype. Together, these data point to a model of high TMB correlating with high proliferation and with both linked to productive antitumor immunity. It had been hypothesized that oncogenes driving sustained proliferation also induce DNA replication stress, which generates genomic instability and presumably increase TMB (35). In summary, TMB provides a mechanistic link between proliferation and antitumor immunity and should be investigated in future studies. Furthermore, our analysis shows enrichment of potential immune checkpoint targets in high-proliferation breast cancer, including PD-1, Lag3, IDO, and PD-L1 elevation.

One of the main goals of this study was to provide methods and a framework for robust identification of spatial biomarkers. Using external cohorts to validate biomarkers discovered in our CycIF data increases our confidence in biomarker identification. In ER^+^ breast cancer, we found that increased stromal neighbors of tumor correlated with better prognosis, similar to previous studies showing a survival benefit of high stroma in ER^+^ tumors (36). We found that macrophage proximity to a tumor was associated with a good prognosis in TNBC, which is unexpected given previous publications. Specifically, tumor-associated macrophages (TAMs) (37) were associated with shorter OS in a cohort of patients who are ER^+^ and ER^–^, but the prognostic value of macrophages specifically in TNBC was not investigated. Furthermore, Medrek et al. (38) found that CD68^+^ macrophages in close proximity to tumor cells were not associated with poor survival, but those out in the stroma were, suggesting that it may be difficult to compare our metric of macrophage-tumor neighbors in a 40 mm radius with previous studies and that further investigation is warranted. Finally, numerous immune-related spatial biomarkers, including immune-immune proximity, B cell–T cell proximity, immunoregulatory interactions, isolated lymphocyte abundance, and lymphocyte occupancy, were associated with good prognosis in TNBC, supporting a model of productive antitumor immunity in the triple-negative subtype. Encouragingly, our results for the prognostic value of lymphocyte spatial metrics were similar to those of Wortman et al. (15) and Patwa et al. (18)

We utilized spatial LDA modeling to analyze multicellular neighborhoods of stromal cells surrounding tumor cells. We identified a neighborhood enriched for Vim^+^ fibroblasts that was independently associated with shorter survival in TNBC. Given the high levels of Vim and low levels of α-SMA, these cells may have an inflammatory phenotype similar to cancer-associated fibroblasts (CAFs) that differentiate under TNF-α + IL-1β stimulation (39). Interestingly, TNF-α + IL-1β have been shown to stimulate prometastatic chemokine expression (CXCL8, CCL2, and CCL5) and aggressive characteristics in TNBC cell lines, mediated in part by direct CAF-tumor cell contact in cocultures (40), consistent with proximity between putative poor-prognosis CAFs and tumor cells in spatial LDA neighborhoods.

The limitations of our study include different antibody probes and imaging systems, resulting in different signal/background ratios for biomarkers across platforms. Therefore, our integrated analysis relied on matching annotated clusters across platforms. This introduces uncertainty since our annotations may not correspond to the same cell types in each platform. Some well-defined phenotypes, such as T cells and proliferating cells, are relatively straightforward, and we found high correlation between cell types on adjacent normal tissue slides analyzed on MIBI and CycIF platforms, respectively (Figure 1F and Supplemental Figure 5, B and C). However, variable performance of antibodies, such as anti-ER, for example, could lead to variability in the classification of phenotypes such as luminal ER^+^ versus luminal tumor across platforms. To correct for platform-specific bias in cell types, we binarized patients into high/low expression within each subtype and platform for survival analysis. However, such binarization may not reflect underlying heterogeneity in quantitative biomarker abundance.

Another limitation of our study is the use of 1–2 TMA cores per patient for analysis. It has been shown that a limited number of TMA cores (≤3) is needed to binarize patients into high and low TILs, although a larger number of cores (≥11) is needed to accurately estimate the mean TIL abundance of a full tissue section (41). Our survival analysis relied on binarizing patients; therefore, the use of TMAs may be appropriate in this context. We undertook a limited analysis of large tissue sections from which we selected “virtual TMA” punches from the tumor core and border. We found that tumor and stromal cell type abundances were not markedly different in the core versus border after normalizing for epithelial content (Supplemental Figure 5, D and G–I). Sampling larger tissue areas could improve spatial biomarker performance, although increased heterogeneity in large sections could introduce noise, especially if they include adjacent normal tissue, which we found to have significantly different epithelial and stromal phenotypes compared with the tumor (Supplemental Figure 5, E and F). The optimal balance between the area analyzed in each tissue and number of patients included for estimation of prognostic tumor microenvironment composition and spatial architecture remains an open and important question in the field.

Overall, our spatial analysis supports the utility of spatial information in uncovering novel biomarkers of patient outcome in breast cancer. The tools developed in this study can be used to analyze additional cohorts further to characterize biomarkers in breast cancer and other tumor types.

## Methods

### Sex as a biological variable.

Only female patients were included, as females account for more than 99% of breast cancer (42).

### Patient samples.

Two breast cancer TMAs were provided by Jennifer Pietenpol (VUMC). All samples were collected at time of surgical resection (mastectomy or breast-conserving surgery) at VUMC with the same fixation protocol. JP-TMA1 had 131 cores of approximately 1.2 mm diameter, with duplicate cores from 19 TNBC, 8 HER2^+^, and 36 patients who are ER^+^. Four TNBC patients included on TMA1 received NAC. JP-TMA2 contained a single, slightly larger (~1.4 mm diameter) core from 39 triple-negative tumors and 1 ER^+^/HER2^+^ core. Thirteen of the patients in TMA2 received neoadjuvant therapy. Clinical outcome and clinicopathological information were available for TMA1 and TMA2. The normal tissue TMA was provided by Rosie Sears (OHSU).

### Imaging data generation and sources.

CycIF staining of tumor tissue was completed on JP-TMA1 and JP-TMA2 using our protocol: dx.doi.org/10.17504/protocols.io.23vggn6. Antibodies used for staining are available in Supplemental Table 1. The whole tissue core was imaged using fluorescence microscopy as described (17). MIBI data were previously published by Keren et al. (12), and the images were downloaded from https://mibi-share.ionpath.com/tracker/imageset under the name “Keren et al., Triple Negative Breast Cancer.” Survival and recurrence data were obtained from a second publication by the same group (18) and were downloaded from https://github.com/aalokpatwa/rasp-mibi; commitID: 31f8ba22cd4d881b03603ab65d7bab6fca0a80b4. IMC imaging data were previously published by Jackson et al. (14), and images and clinical data were downloaded from https://doi.org/10.5281/zenodo.3518284

### Image processing.

CycIF tiff images were registered and segmented, and single-cell intensity as well as nuclear size and shape features were extracted as described (17). Nuclear and cell segmentation were run using the Cellpose algorithm (43), which showed visually superior performance on CycIF data compared with a watershed algorithm (Supplemental Figure 17A). Nuclear and cell segmentation masks were matched using mplexable, enabling subtraction of nuclear mask from cell mask to obtain segmentation of the cytoplasm (Supplemental Figure 17B).

MIBI and IMC images were downloaded as multipage open microscopy environment (OME) tiffs. Hot pixels (13) were detected by identifying pixels that were 10 SDs above a median filtered image with a 2 × 2 pixel kernel size. Hot pixels were set to the median filter values, and resulting images were saved as tiffs for downstream feature extraction. For nuclear segmentation preprocessing, DNA images were rescaled by clipping at the third and one-and-a-half times the 99.999 quantile. The γ value was adjusted by 0.6 in MIBI data and 0.4 in IMC data to enhance dimly stained nuclei. A 2-channel nuclear plus cytoplasm image was generated for cell segmentation. For MIBI cytoplasm segmentation preprocessing, the β-catenin, Vim, CD45, and CD31 channels were combined into a maximum intensity projection image and the γ value was adjusted by 0.6. For IMC cytoplasm segmentation preprocessing, Ecad, Vim, CD44, and CD45 were combined into a maximum intensity projection image, and γ was adjusted by 0.4. Chambolle total variation denoising, implemented in scipy (44), was used to smooth out pixelated nuclear and cytoplasmic projection images (weight = 0.1, except weight = 0.05 for IMC cytoplasm). All parameters were selected by testing segmentation results at https://www.deepcell.org/predict and https://www.cellpose.org/ (Supplemental Figure 17, C and D). Skipping either nuclear or cytoplasmic Chambolle total-variation de-noising resulted in failure of deep learning–based algorithms on the IMC data (Supplemental Figure 17E). Mesmer segmentation (45) performed better than Cellpose (43) in IMC data due to improved detection of dim nuclei, likely due to the incorporation of cytoplasmic staining in the nuclear segmentation model (Supplemental Figure 17D). Cellpose was successful in CycIF images, which had brighter DNA staining. For IMC and MIBI data, nuclear and cellular segmentation were performed on preprocessed segmentation images using Mesmer (45). Matching of cell IDs in the nuclear and cell masks was done with mplexable (17), with cell masks relabeled to match the ID of the nucleus to which they had most overlap. Cytoplasm masks were calculated by subtracting the nuclear mask from the matching cell mask. Nuclear and cytoplasmic mean intensity, nuclear size and shape features, and nuclear centroid coordinates were extracted with mplexable (17).

Mesmer segmentation was compared with the watershed-based segmentation originally published by Jackson et al. (14). The cell counts across the 2 methods had a Pearson correlation of 0.98 (Supplemental Figure 18A). Visual examination of ROIs with discordant cell numbers revealed that Mesmer segmentation performed better in tissues with necrosis and high background noise in the DNA channel (Supplemental Figure 18, B–D).

### Image quality control.

In IMC data, artifacts include nonspecific background staining, necrotic regions, and bright antibody aggregates. IMC data were collected from small ROIs (600 × 600 µm) within TMA cores, and some samples annotated as ER^+^ tissues did not show any ER^+^ staining in the ROI. Therefore, quality control (QC) was performed on ER-stained images, a marker noted to exhibit nonspecific background staining on the IMC platform (46). QC images of ER staining were generated and sorted in a blinded fashion into negative and positive for nuclear-specific staining (Supplemental Figure 19A). Only ROIs from clinically annotated patients who are ER^+^ and classified as ER^+^ during QC, or ROIs from ER^–^ patients and classified as ER^–^, were used for analysis (Supplemental Figure 19B). Samples that passed ER QC did not have significantly different grade, PR status, TMA block, age of specimen, age of patient or tumor size compared with those that failed QC (Supplemental Figure 19, C and D). There were no significant survival differences between QC passed versus failed tumors from patients who were ER^+^, TNBC, or ER^+^HER2^+^ (Supplemental Figure 19E). Additional QC steps included the following: necrotic regions were manually circled using the napari (47) image viewer and excluded, and bright aggregates in the CD3 channel were excluded by removing cells above a threshold set at the intensity of CD3^+^ cells showing an appropriate membranous staining pattern.

In the CycIF data, imaging artifacts included autofluorescence (AF), nonspecific background, floating tissue, and tissue loss. Background AF images were obtained halfway through CycIF data collection, and these images were scaled by exposure time in each round of staining and subtracted from the AF488, AF55, and AF647 channels using mplexable (17). Feature extraction was performed on AF subtracted images. Areas of floating tissue, air bubbles, or necrotic regions were manually circled using the napari (47) image viewer and excluded. Nonspecific background staining was removed by setting intensity thresholds for selected markers and by subtracting those values from extracted data. The PD-1 antibody had bright aggregates that were excluded with an upper threshold. Tissue loss was detected by cells that lacked DAPI staining in the last round of imaging, and these cells were excluded.

In all 3 platforms, additional artifacts caused by floating tissue or imaging problems (e.g., dark or bright bands across IMC and MIBI, perhaps caused by problems with the rastering process) were detected through unsupervised clustering and visual inspection of clusters on the images. Clusters composed of artifacts showed atypical, very bright or dim staining in many channels; formed distinct artifact clusters; and were removed.

### Single-cell phenotyping.

Cell types were defined in 2 ways: manual gating and unsupervised clustering. Unsupervised clustering was conducted using the scanpy (19) software. Single-cell mean intensity values were selected from either the nucleus or cytoplasm masks for each marker, depending on expected subcellular distribution. Since the CycIF and IMC platforms had more marker and breast cancer subtype overlap than the MIBI panel (Figure 1B and Supplemental Figure 20), 20 matching markers were selected for clustering in these datasets as well as selected markers for immune, epithelial, and fibroblast subsets (ColI, CD4, CD8 in CycIF, and FN, pan-cytokeratin [panCK] in IMC). For MIBI data, all available markers were used for clustering. Additionally, the nuclear area feature was used for clustering. Each marker was divided by its SD, without zero-centering, and clipped above 20 SD. A UMAP embedding was generated using 30 k-nearest neighbors and clustered using the Leiden community detection algorithm (48). The Leiden resolution parameter was selected that resulted in 20–25 clusters. Each cluster was annotated and categorized as epithelial, endothelial, fibroblast, immune, or stromal. Some clusters were composed of multiple expected cell types, and these were manually split; for example, the CD44^+^ cluster was split into CD44^+^ tumor and CD44^+^ stroma based on manual gating results (described below).

We then performed manual gating to verify our annotated-cluster cell type. A threshold was set for each gating marker based on the expected pattern of positive staining in images. Fibroblasts were defined as positive for 1 or more of Vim, FN, or ColI. Epithelial cells were defined as positive for 1 or more of Ecad, cytokeratin, or β-catenin. Endothelial cells were defined as CD31^+^. Immune cells were defined as CD45^+^. Stromal cells were defined as all nonfibroblast, nonendothelial, nonepithelial, nonimmune segmented nuclei.

### Patient subtyping.

Epithelial and stromal subtypes were determined by unsupervised clustering of patients based on the fraction of epithelial or stromal cell types within each compartment, respectively. Cell types representing greater than 2%–4% of the total cell population in the respective tissue compartment were used for clustering. This cutoff was chosen to exclude rare cell types that may represent method-specific artifacts. Cell fractions were normalized across platform using standard scaling. Unsupervised clustering of patients was performed using the Leiden algorithm implemented in scanpy (19). For epithelial subtypes, the resolution of clustering was selected to minimize differences between the platforms (Figure 2). For stromal subtypes, we selected the minimum number of clusters needed to separate T cells from other clusters (*k* = 6).

### Survival analysis.

The CycIF TMA1 dataset was used as a discovery dataset to determine the quantile separating high and low abundance of each cell type or spatial metric that was most predictive of survival. Three quantiles were tested: 0.33 (e.g., split patients into one-third low and two-thirds high), 0.5, and 0.66. The most prognostic cutoff value was selected for each cell type, and for cell types having prognostic value (α < 0.05), these cutoffs were applied in the validation dataset, which included CycIF TMA2, MIBI, and IMC samples. Since overall cell type fractions differed between platforms and subtypes (Figure 1), high and low values were determined relative to other samples from the same platform and subtype, using cutoffs from the discovery cohort. In the validation cohort, the log-rank test *P* values were corrected for multiple testing using the Benjamini-Hochberg method. For biomarkers with FDR < 0.1, multivariable CPH modeling was used to combine imaging biomarkers with patient age, tumor size, and clinical stage to test if they were independently prognostic. Collectively, 89 TNBC and 160 patients who are ER^+^ had these additional clinical parameters (not available in the MIBI dataset).

### Spatial analysis.

Spatial distributions of cells were calculated as follows. For analysis of cellular neighbors (11) and homotypic/heterotypic interactions (13), each cell’s neighbors within a 40 μm radius were counted. For tumor-immune mixing score, a 25 μm radius was selected to replicate Keren et al. (12) For lymphocyte clusters, a 20 μm radius was used, and for lymphocyte occupancy, 10–300 μm grid squares were used, at 10 μm steps, both selected to replicate Wortman et al. (15). Voronoi tessellation was used to replicate spatial interactions as defined in Patwa et al. (18). Ripley’s L (a density-normalized measure of clustering) and the multitype K function (Kcross; a density-normalized measure of 2 cell types colocalization) and G function (Gcross, a measure of 2 cell types colocalization) were calculated using spatsat (23) with a radius of 50 μm. Spatial LDA analysis was done using spatial-LDA (24), using the default radius of 100 μm and 8 topics. Shorter distances of ~25 μm may be interpreted as cells nearly or directly touching, while 100 μm represents a distance at which oxygen, nutrients, and potentially other molecules diffuse in tissues (49). Survival analysis was done as described above, using the CycIF TMA1 dataset as a discovery cohort and the other datasets as the validation cohort. For previously published biomarkers, the validation cohort included patients not used in developing those biomarkers. Specifically, the tumor-immune mixing score, developed using the MIBI cohort, was validated with the CycIF and IMC cohorts. Immunoregulatory interactions, also developed using the MIBI dataset, were validated in the CycIF cohort only, since the IMC panel lacked immunoregulatory markers. Lymphocyte clustering, lymphocyte occupancy, and heterotypic neighbor biomarkers were initially developed in external cohorts, so all samples were included in the validation cohort (and no discovery cohort was used).

### Statistics.

For comparison of continuous variables, 2-sided Pearson correlation was used. For categorical correlations, 2-sided Mann-Whitney *U* test (2 groups) or Kruskal-Wallis H-test (3 or more groups) were used, with Tukey’s HSD correction for pairwise comparisons between groups. Two-way χ^2^ analysis was used for categorical data, with Bonferroni correction for pairwise comparisons between groups. Survival curves were estimated with Kaplan-Meier analysis, and the log-rank test was used to calculate significance. CPH modeling was used to estimate HRs and *P* values in multivariable survival models. *P* < 0.05 was considered significant. When multiple variables were tested for survival, Benjamini-Hochberg correction was applied in the validation cohort. FDR < 0.05 was considered significant, except with spatial LDA neighborhoods, where FDR < 0.1 was considered significant.

### Study approval.

Samples were obtained with written informed consent; sample collection complied with all relevant ethical regulations and was approved under Vanderbilt IRB protocol no. VICC BRE03103. Use of human samples for our study at OHSU was approved under IRB protocol no. STUDY00016712.

### Data availability.

Spatial and survival analysis code; CycIF, MIBI, and IMC processing pipelines, single-cell phenotyping pipelines and precomputed data are available here: https://github.com/engjen/cycIF_TMAs Supporting data values and antibody information is available in our Supporting Data Values file**)**. Images and data files larger than 10 MB are available here: https://www.synapse.org/#!Synapse:syn50134757/

## Author contributions

JRE, SLG, and KC conceived of the project. MES, ABC, PGE, and JAP collected patient tissues and clinical outcome data and constructed the TMAs. ZH performed staining and imaging experiments. JRE and EB did single-cell image processing. CRW, TR, and MA generated MIBI data on normal tissues. JRE ran the analysis, drafted the manuscript, and prepared the figures. SLG, KC, EB, ABC, RCS, and JAP edited the manuscript.

## Supplementary Material

Supplemental data

Supplemental table 1

Supporting data values

## Version 1 01/14/2025

In-Press Preview

## Version 2 02/10/2025

Electronic publication
