# Supplementary material for: Highly multiplexed imaging reveals prognostic immune and stromal spatial biomarkers in breast cancer
Source: JCI Insight. 2025 Jan 14;10(3):e176749. doi: 10.1172/jci.insight.176749 (PMC11948582; doi:10.1172/jci.insight.176749)
Supplement: Supplemental data [file jciinsight-10-176749-s166.pdf]

Supplement to Eng J. et al. Highly multiplexed imaging reveals prognostic immune and stromal spatial biomarkers in breast cancer.

LIST OF SUPPLEMENTAL FIGURES

Supplemental Figure S1. Stage, grade, neoadjuvant chemotherapy and reproducibility.
Supplemental Figure S2. Single Cell Analysis of CyCIF data.
Supplemental Figure S3. Single Cell Analysis of IMC data.
Supplemental Figure S4. Single Cell Analysis of MIBI data.
Supplemental Figure S5. Correlation of gating and cell types across platforms, effect of location of TMA core.
Supplemental Figure S6. Normalization of epithelial fractions across platforms.
Supplemental Figure S7. Prognostic Value of Epithelial Subtypes.
Supplemental Figure S8. Prognostic Value and Clinical Subtype Correlation of Stromal Subtypes.
Supplemental Figure S9. Single Variable Prognosis in separate cohorts.
Supplemental Figure S10. Prognostic value of proliferation and T cell abundance.
Supplemental Figure S11. Prognostic value of tumor immune spatial metrics.
Supplemental Figure S12. Prognostic value of immunoregulatory, lineage and functional interactions, co-expression.
Supplemental Figure S13. Correlation of Spatial Metrics and tissue composition.
Supplemental Figure S14. Spatial LDA Tumor Neighborhoods.
Supplemental Figure S15. Neighborhoods defined by directly clustering the cell counts.
Supplemental Figure S16. Correlation of tumor neighborhoods and tissue composition.
Supplemental Figure S17. De-noising and Segmentation Optimization.
Supplemental Figure S18. Mesmer versus Watershed Segmentation.
Supplemental Figure S19. ER Quality Control.
Supplemental Figure S20. Comparison of breast cancer MXI panels and dataset sizes.

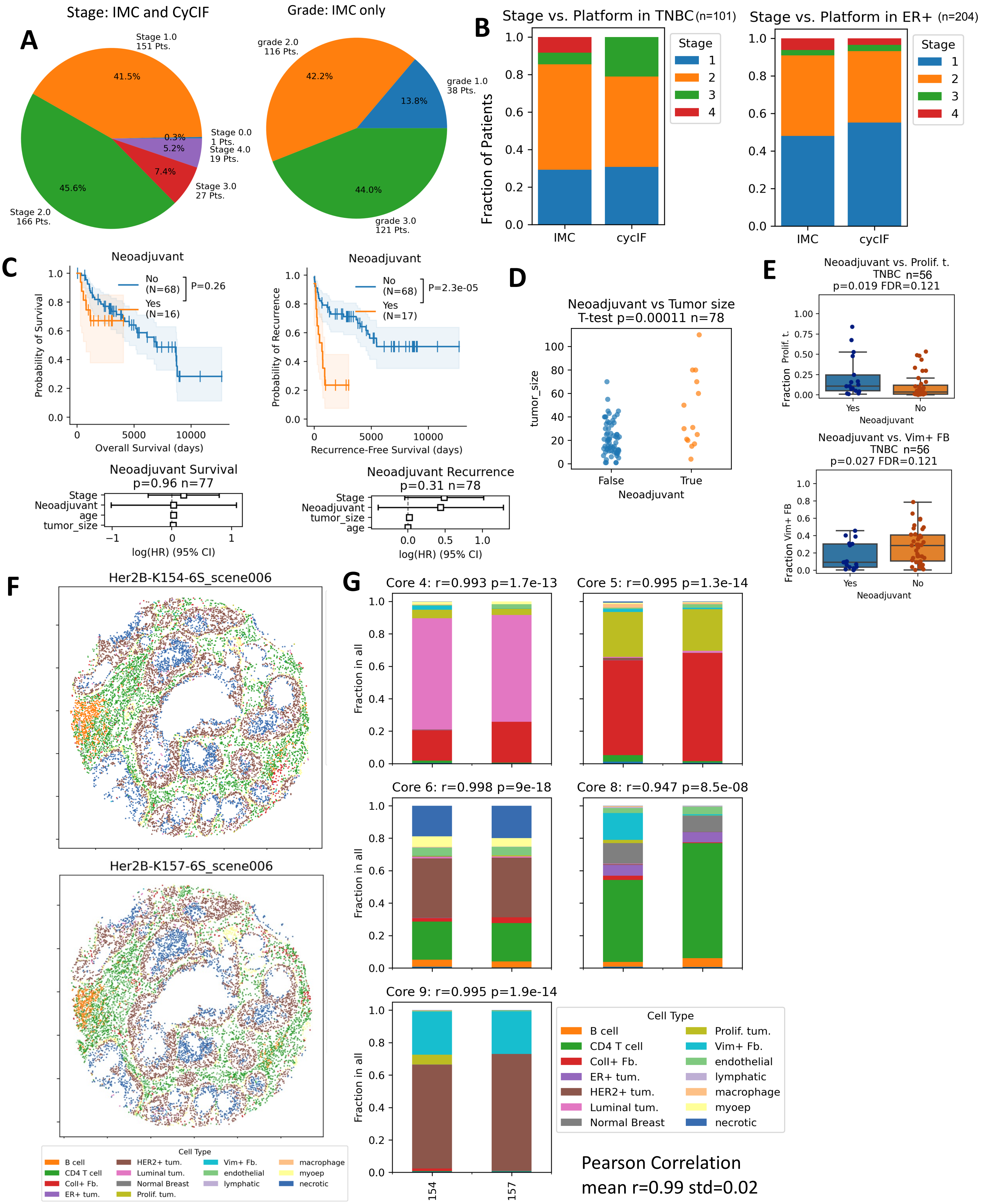

S1. Stage, grade, neoadjuvant chemotherapy and reproducibility.

A. Pie charts of fraction and number of patients of each stage (left) and grade (right) for datasets with clinical information. B. Fraction of patients of each stage in each dataset/subtype. C. Top: Kaplan-Meier analysis of overall survival (OS, left) and recurrence free survival (RFS, right) for patients with or without neoadjuvant chemotherapy (NAC). P-value from log-rank test. Bottom: Multivariable Cox Proportional hazard modeling of OS (left) and RFS (right). P-value of neoadjuvant variable and n number of patients in panel title. D. Tumor size versus neoadjuvant status, p-value in panel title from two tailed T test. E. Fraction of cells in tumor (top) or stromal (bottom) compartment in patients receiving NAC versus not. P-value from Mann Whitney U test, FDR corrected for multiple cell types tested with the Benjamini Hochberg method. F. Spatial localization of cell types in TMA cores from serial tissue sections stained with CycIF on different dates. G. Fraction of each cell type in each core of TMAs from (f), Pearson correlation r- and p-value in panel titles. A-E. N number of patients indicated in panel titles.

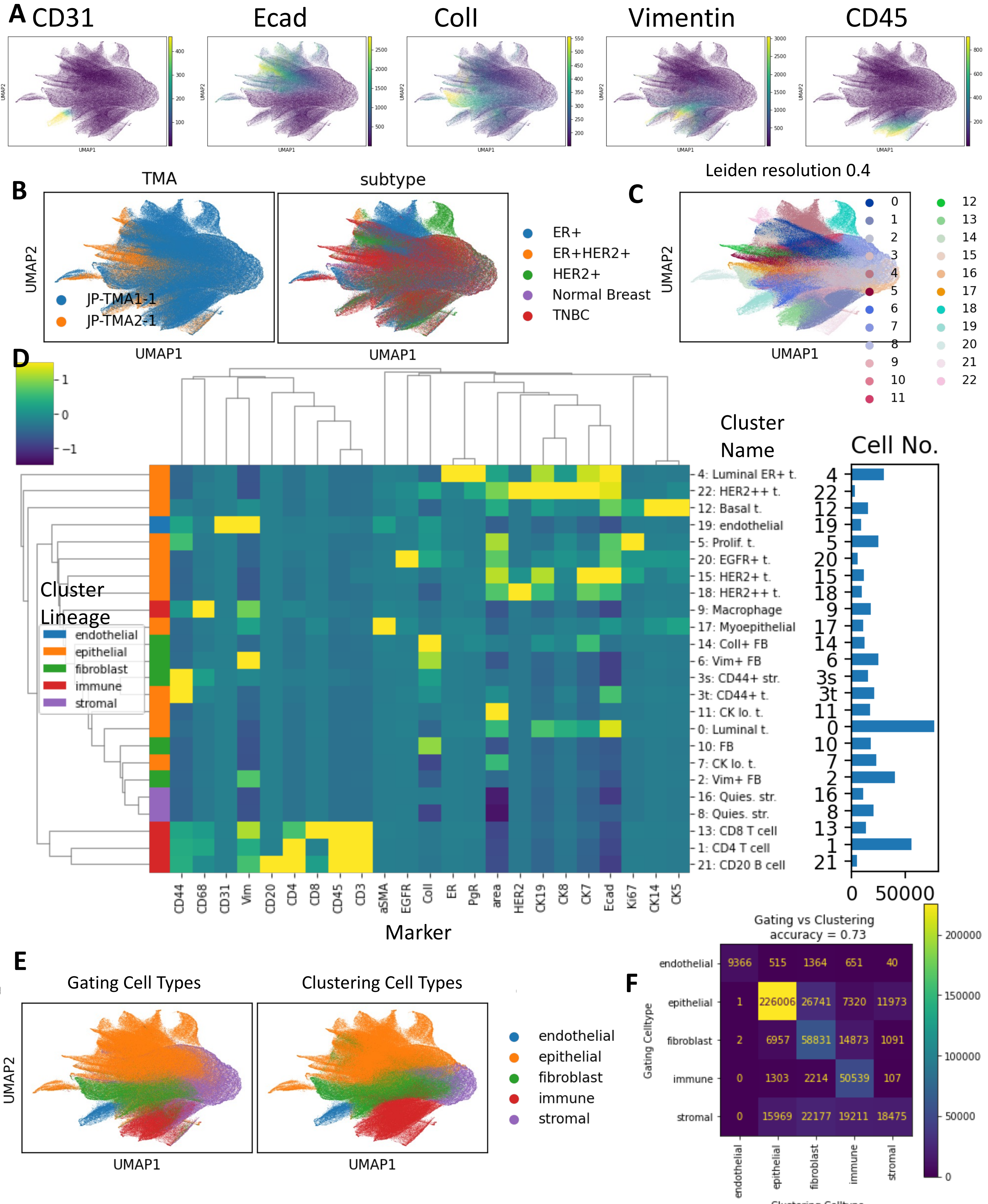

S2. Single Cell Analysis of CyCIF data

A. Single-cell segmentation and feature extraction were done with mplexable. A UMAP embedding was generated based on single-cell mean intensity values (30 k-nearest neighbors). The UMAP is colored by cell lineage markers CD31, endothelial, E-cadherin (Ecad) epithelial, collagen I (ColI) and vimentin, fibroblast, and CD45, immune. B. UMAP colored by TMA (left) and breast cancer subtype (right). C. Unsupervised clustering with the Leiden algorithm (resolution 0.4) resulted in 23 cell types. D. Heatmap of mean fluorescence intensity of each marker in CyCIF cell type clusters. Twenty-two markers and one morphology feature (nuclear area) were used for clustering. Cell types were annotated as endothelial, epithelial, fibroblast, immune or stromal (left color bar on heatmap) and named based on marker expression (right labels on heatmap). E. Manual gating of the markers in (a) were used to determine cell types (left) separately from the Leiden annotated cell lineages (right). F. Confusion matrix of gating-based versus clustering-based cell lineages shows 73% accuracy.

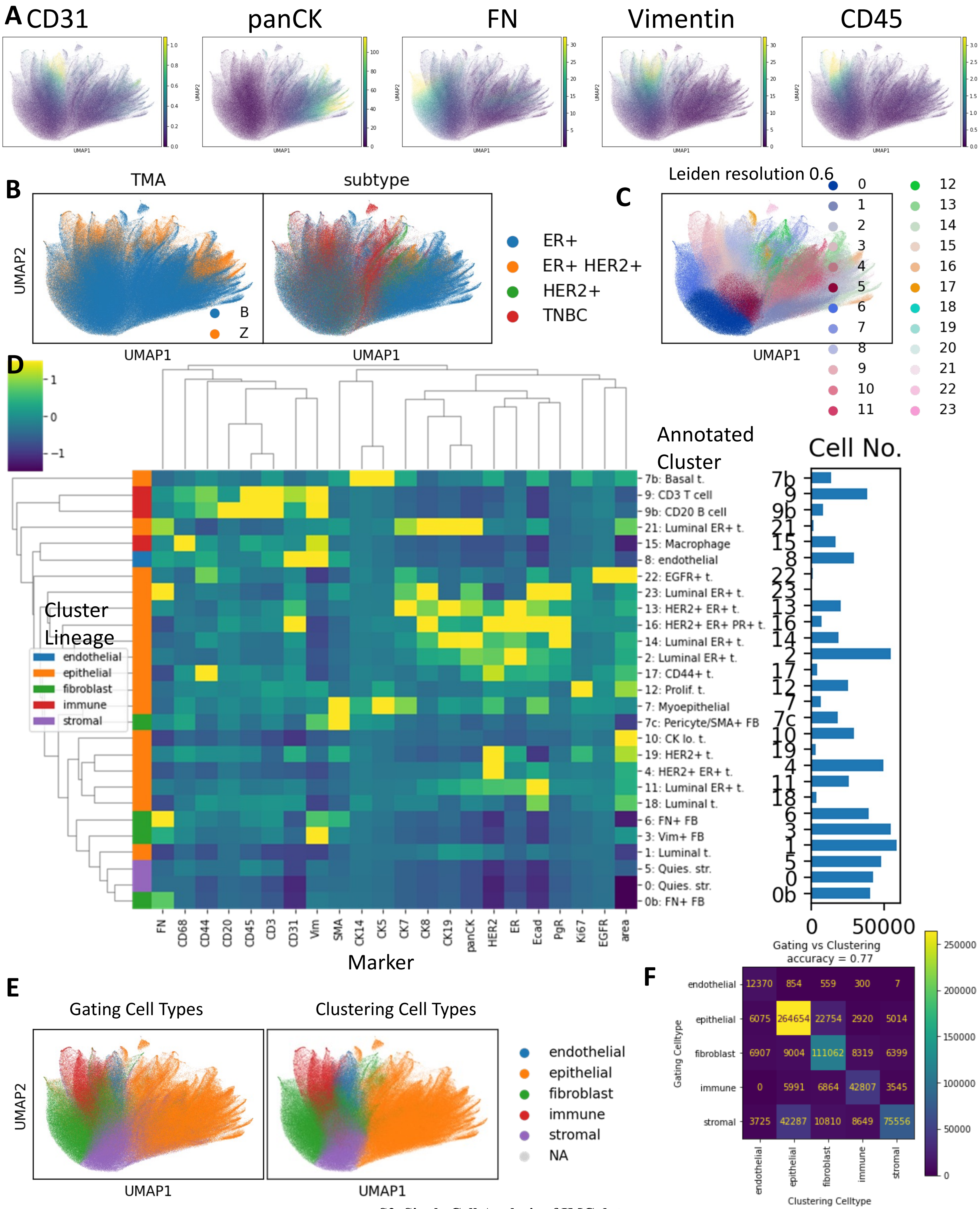

Gating \ Clustering	endothelial	epithelial	fibroblast	immune	stromal
endothelial	12370	854	559	300	7
epithelial	6075	264654	22754	2920	5014
fibroblast	6907	9004	111062	8319	6399
immune	0	5991	6864	42807	3545
stromal	3725	42287	10810	8649	75556

S3. Single Cell Analysis of IMC data

A. Single-cell segmentation and feature extraction were done with mplexable. A UMAP embedding was generated based on single-cell mean intensity values (30 k-nearest neighbors). The UMAP is colored by cell lineage markers CD31, endothelial, pan-cytokeratin (panCK) epithelial, fibronectin (FN) and vimentin, fibroblast, and CD45, immune. B. Two TMAs (left) and four subtypes (right) were clustered together for cell typing. C. Unsupervised clustering with the Leiden algorithm (resolution 0.6) resulted in 25 cell types. D. Heatmap of mean fluorescence intensity of each marker in IMC cell type clusters. Twenty-one markers and one morphology feature (nuclear area) were used for clustering. Cell types were annotated as endothelial, epithelial, fibroblast, immune or stromal (left color bar) and named based on marker expression (right). E. Manual gating of the markers in (a) were used to determine cell types separately from the Leiden annotated cell types. F. Confusion matrix of gating-based versus clustering-based cell lineages shows a 77% agreement.

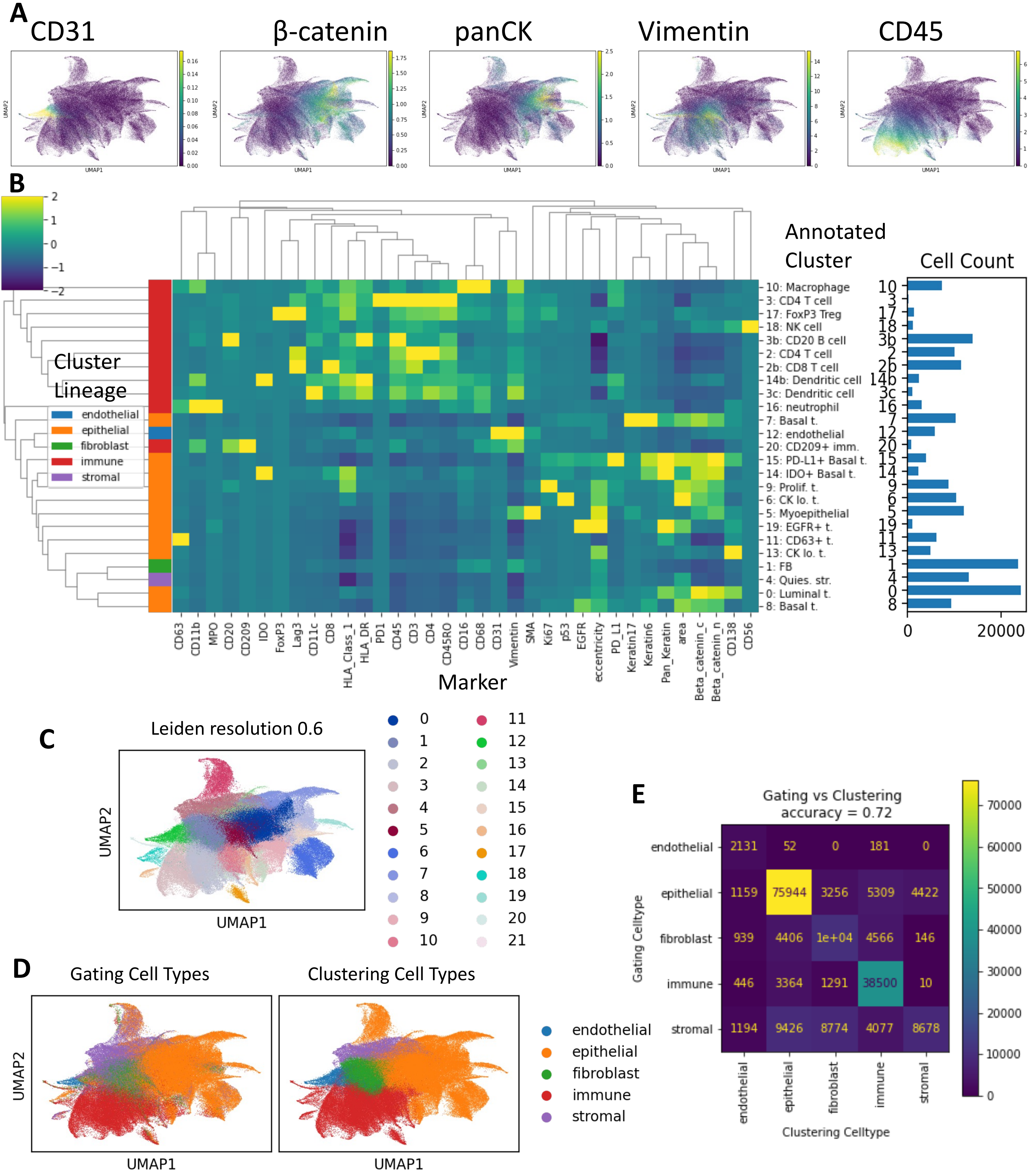

S4. Single Cell Analysis of MIBI data

A. Single-cell segmentation and feature extraction were done with mplexable. A UMAP embedding was generated based on single-cell mean intensity values (30 k-nearest neighbors). The UMAP is colored by cell lineage markers CD31, endothelial, β-catenin and pan-cytokeratin (panCK) epithelial, vimentin, fibroblast, and CD45, immune. B. All samples were from a triple-negative breast cancer TMA. Thirty-three markers and one morphology feature (nuclear area) were used for clustering. Cell types were annotated as endothelial, epithelial, fibroblast, immune or stromal (left color bar) and named based on marker expression (right). C. Unsupervised clustering with the Leiden algorithm (resolution 0.6) resulted in 22 cell types. D. Manual gating of the markers in (A) were used to determine cell types separately from the Leiden annotated cell types. E. The gating-based versus clustering-based cell types had a 72% agreement.

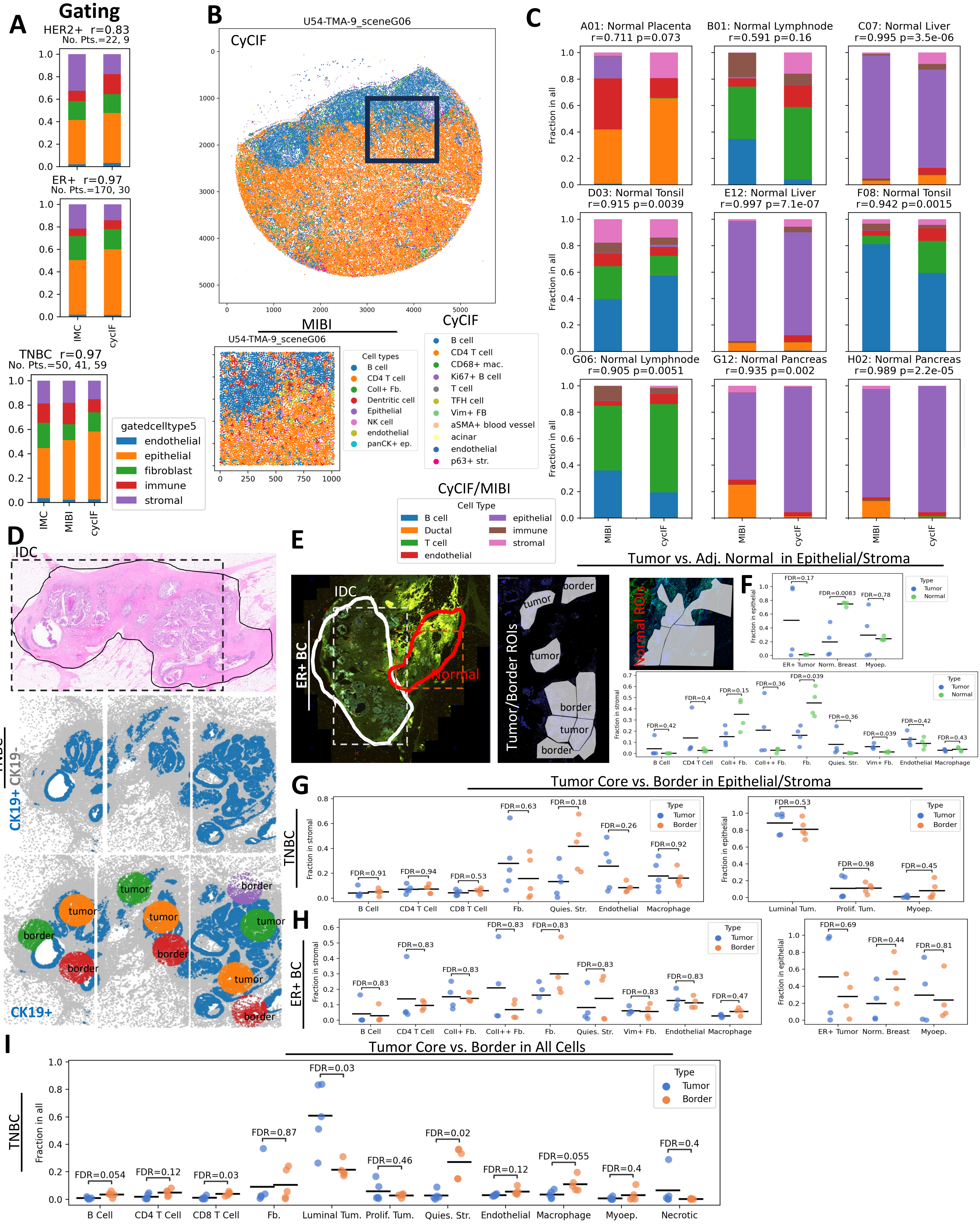

S5. Correlation of gating and cell types across platforms, effect of location of TMA core.

A. Cell lineage fraction of total cells per subtype, per platform using manual gating to determine lineage. Pearson's correlation between platforms (r=0.xx) and number of patients for each subtype and platform shown in panel title. B. CyCIF and MIBI cell types in lymph node from adjacent sections of the same TMA. MIBI ROIs are 500 x 500 µm. C. Fraction of CyCIF and MIBI cell types in each core from adjacent sections of the same TMA containing normal tonsil, lymph node, liver, pancreas and placenta. Pearson's correlation r and p-value in panel title. D-E. CyCIF data from full tissue sections of TNBC (D) and ER+ (E) tumor, respectively, showing selected ROIs from within the tumor core (i.e. CK19+ cells), on the tumor/stroma border, and in the adjacent normal tissue. F-I. Fraction of each cell type in indicated compartment (epithelial, stromal or all cells) for tumor (blue) vs. normal (green) and tumor vs. border (orange). Each dot is one ROI from D-E. FDR corrected p-values from two-tailed t-test corrected for multiple cell types w/ Benjamini-Hochberg method.

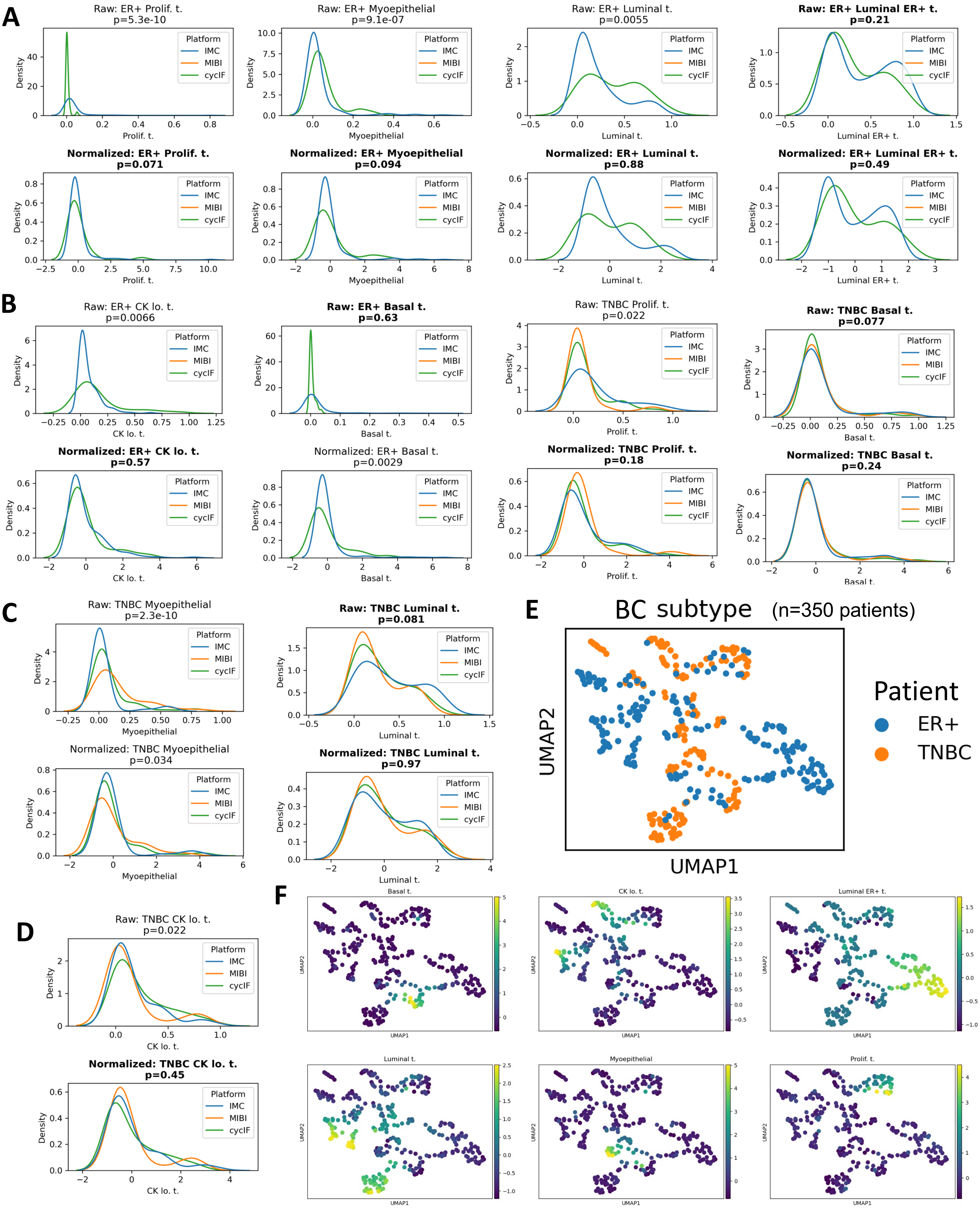

S6. Normalization of epithelial fractions across platforms.

A-D. Kernel density estimates of fraction of epithelial cells of each phenotype before (top) and after (bottom) z-score normalization by platform. P-value in panel title is significant difference between platforms by Kruskal-Wallis H-test. Title text is bolded for p-values >0.05 indicating normalization resulted in no significant differences in median abundance of cell types between the platforms. E. UMAP embedding of patients by fraction of epithelial cell types in all tumor cells, colored by clinical subtype. F. UMAP embedding from (E), colored by marker abundance.

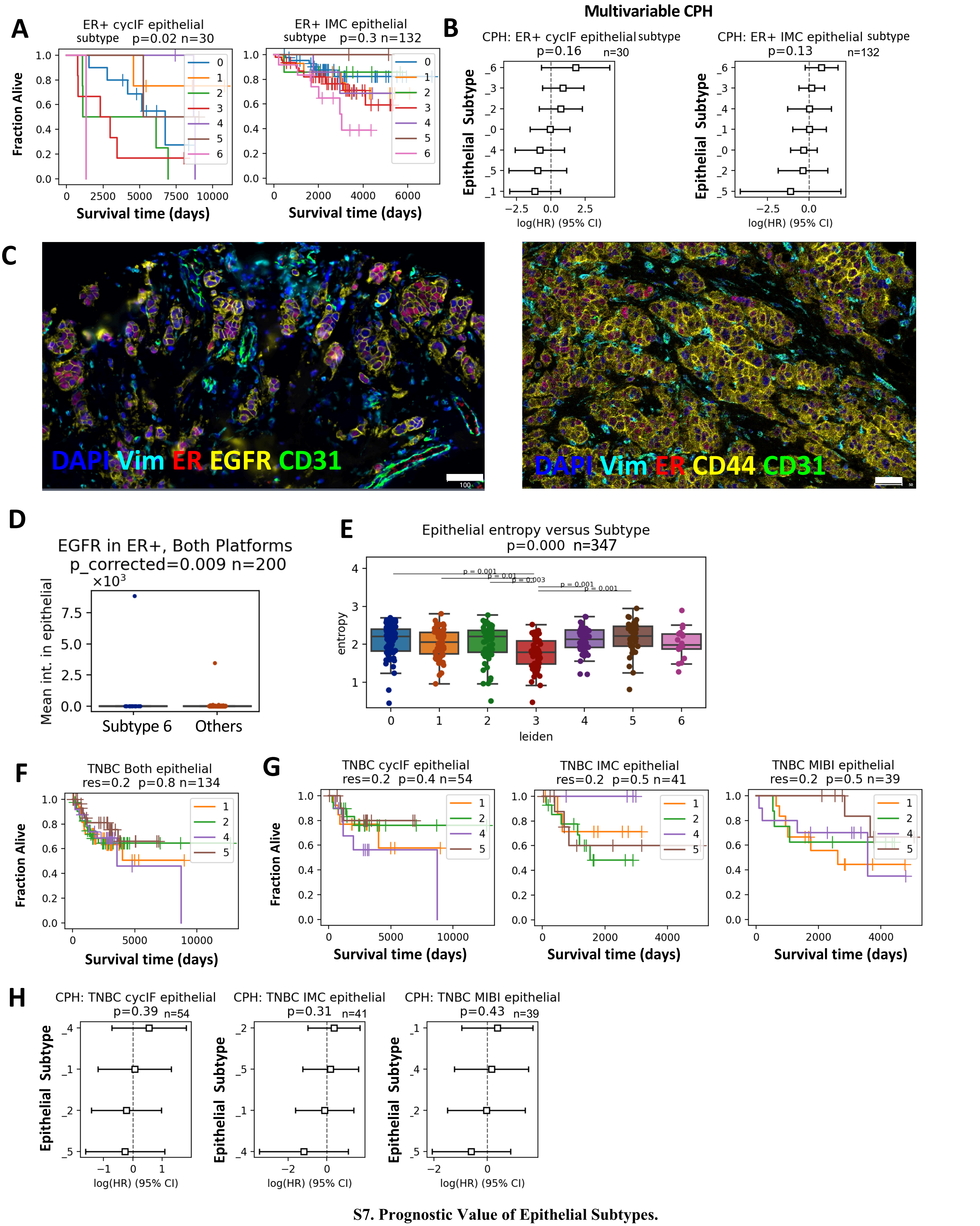

S7. Prognostic Value of Epithelial Subtypes.

A. Kaplan-Meier (K-M) curves (p-value from log-rank test) comparing overall survival (OS) in epithelial subtypes in ER+ tumors, by platform. B. Cox proportional hazard (CPH) modelling of epithelial subtypes versus overall survival in ER+, by platform. C. Example images of subtype 6 in ER+ tumors from the cycIF (left) and IMC (right) cohorts. scale bar=100 µm. D. Mean intensity of selected markers of ER+ patients in poor-prognosis subtype 6 versus other ER+ patients. p-values obtained from t-tests and corrected for multiple markers with the Benjamini–Hochberg method. E. Shannon entropy of patients' epithelial phenotypes in each epithelial subtype. F. K-M OS curves of epithelial subtypes in all TNBC. G. K-M OS curves of epithelial subtypes in TNBC, by platform. H. CPH modelling of epithelial subtypes OS in TNBC, by platform. A-B, F-H. Platform, N number of patients and p-value shown in panel title.

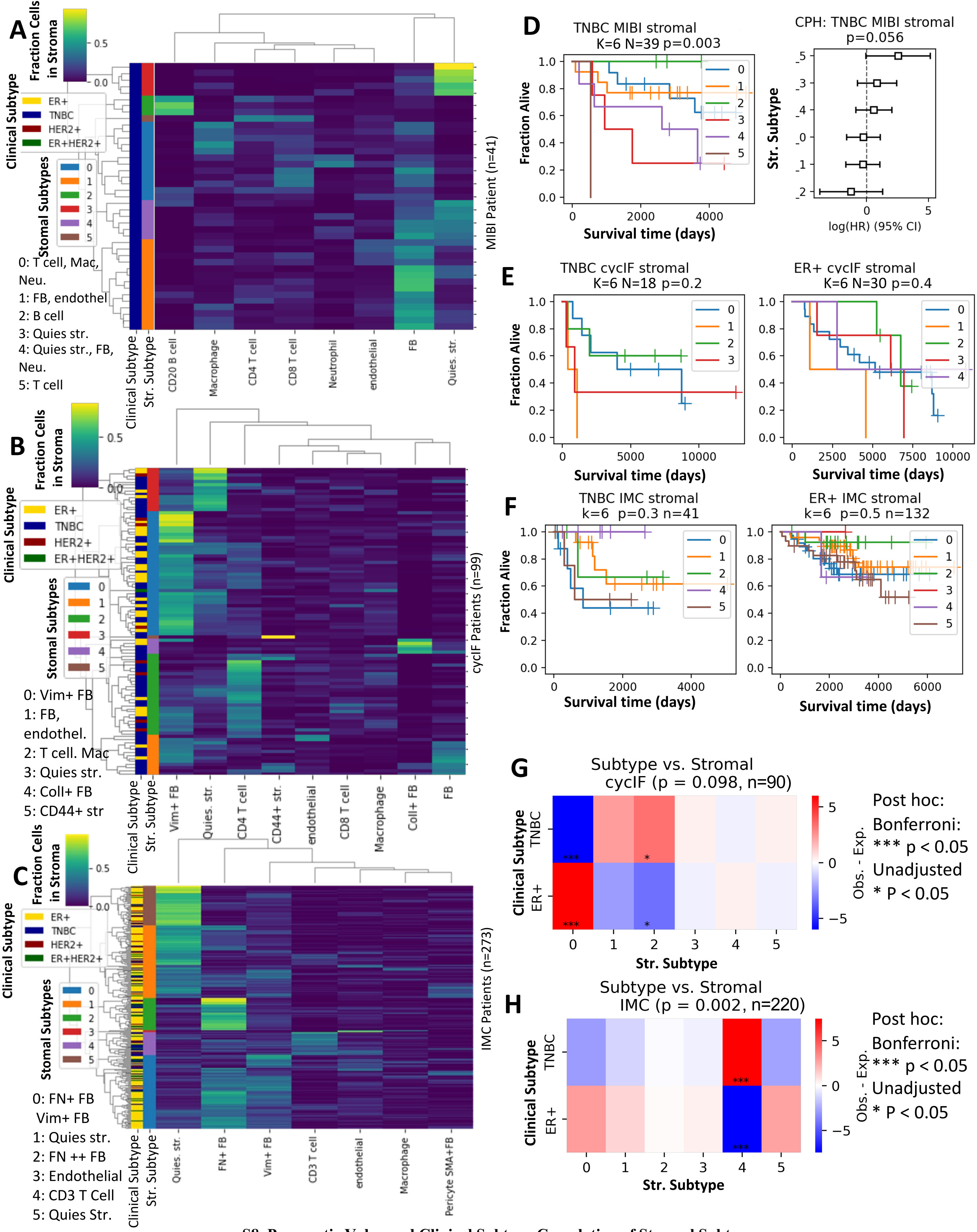

S8. Prognostic Value and Clinical Subtype Correlation of Stromal Subtypes.

A-C. All patient tissues from each platform were hierarchically clustered based on the fraction of the common stromal cell types (>2%) in all stromal cells, selecting k=6 stromal subtypes, annotated on lower left of panel. Platform and number of patients indicated on right panel y-axis. D. MIBI Kaplan-Meier curves (p-value from log-rank test) and Cox proportional hazard (CPH) models comparing OS in stromal subtypes, n=39 patients. E. CyCIF Kaplan-Meier curves comparing OS in stromal subtypes (p-value from log-rank test) n=18 TNBC, 30 ER+ patients from TMA1. F. IMC Kaplan-Meier curves comparing OS in stromal subtypes (p-value from log-rank test) n=41 TNBC, 132 ER+ patients. G-H. Observed minus expected number of patients for each clinical subtype versus stromal subtype for CyCIF (G) and IMC (H). Overall p-value given in title (Chi-squared), total patients and pairwise Bonferroni adjusted (***) and unadjusted (*) p-values < 0.05 marked on heatmap cells.

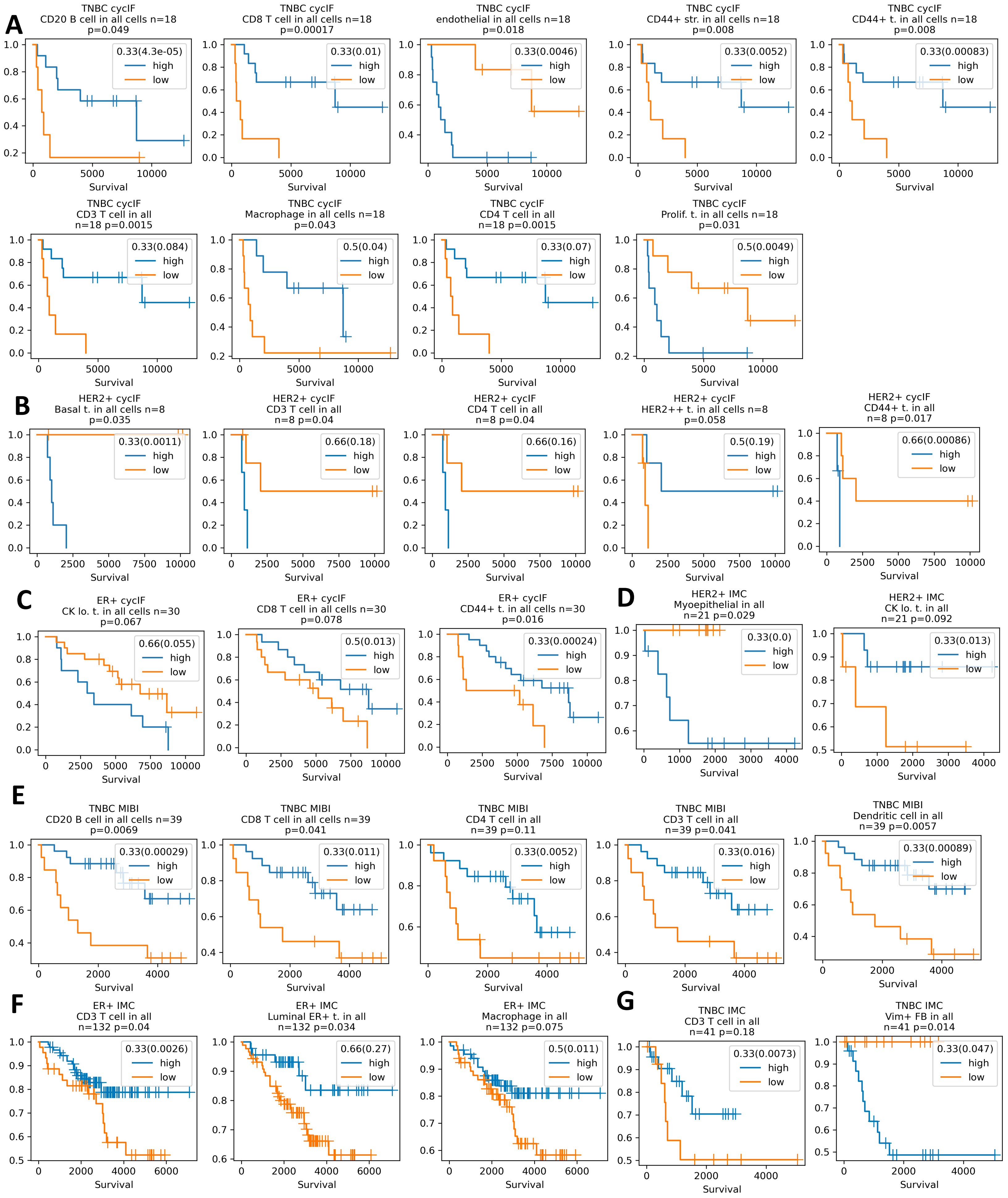

S9. Single Variable Prognosis in separate cohorts.

A-C . Kaplan-Meier (K-M) curves of high/low abundance of various cell types versus OS in CyCIF cohort for TNBC (A), HER2+ (B) and ER+ (C). D-G. Kaplan-Meier (K-M) curves of high/low abundance of cell types versus OS in IMC and MIBI cohorts. A-E. Cut-off values (tertiles of median) given in K-M legends. K-M p-values derived from log-rank test and given in figure panels, along with subtype and n number of patients. y-axis is fraction of patients alive and x-axis is overall survival in days.

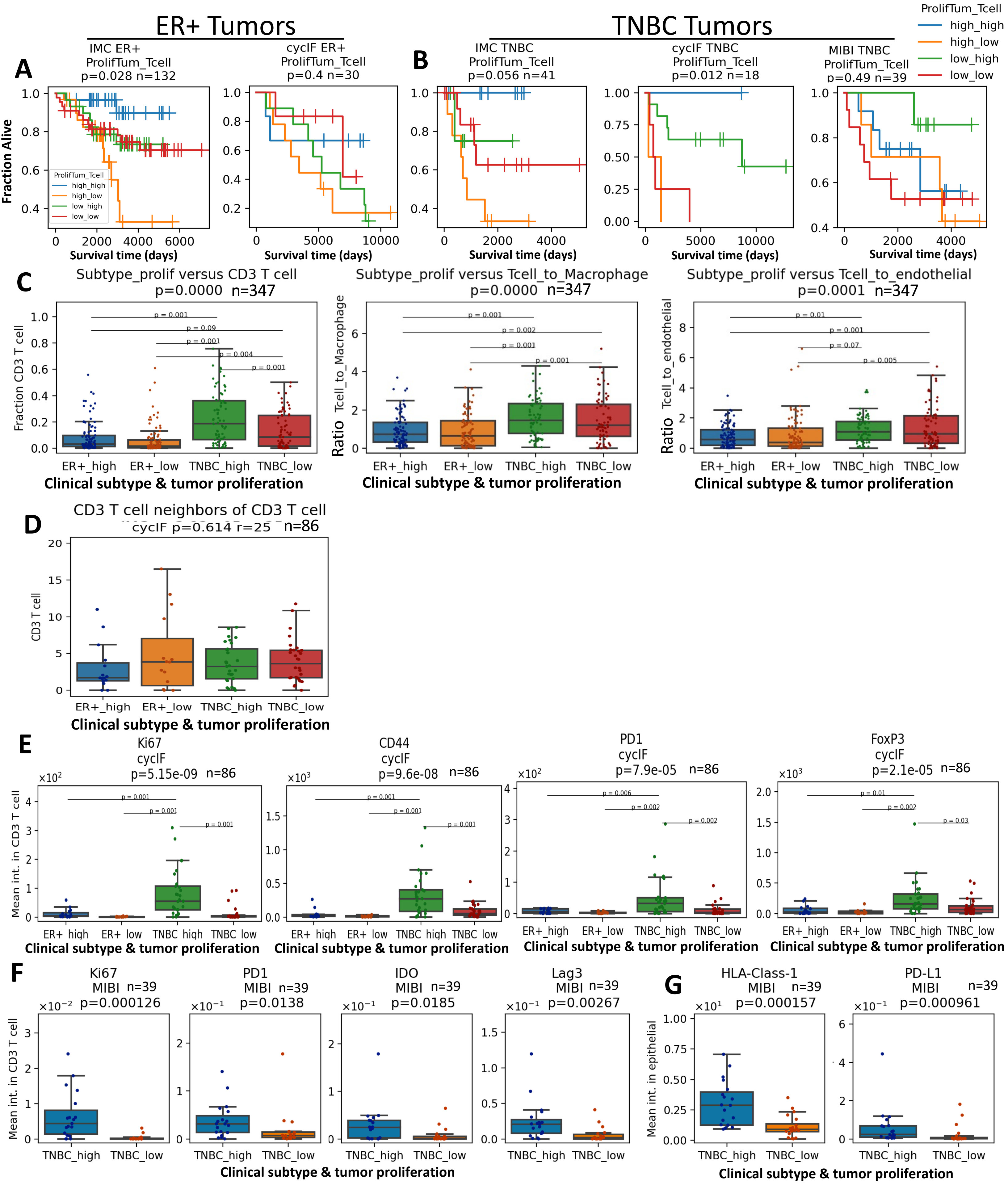

S10. Prognostic value of proliferation and T cell abundance.

A-B. Kaplan-Meier analysis of overall survival in each subtype split by median tumor proliferation and median CD3 T cell abundance from separate platforms' ER+ (A) and TNBC patients (B). platform and p-value (log-rank) given in panel title. C. Fraction of T cells and ratio of T cells to macrophages and T cells to endothelial cells in tissues from high and low proliferation ER+ or TNBC tumors in the CyCIF and IMC cohorts combined. D. Mean number of T cell neighbors of T cells within a 25 µm radius in tissues from high and low proliferation ER+ or TNBC tumors in the CyCIF cohort. E. Ki67, CD44, PD1 and FoxP3 intensity in T cells indicating proliferation, memory/effector, checkpoint and regulatory function in tissues from high and low proliferation ER+ or TNBC tumors in CyCIF cohort. F. Ki67, PD1, IDO and Lag3 intensity in T cells indicating proliferation and checkpoint function in tissues from high and low proliferation TNBC tumors in MIBI cohort. G. HLA-Class-1 and PD-L1 in epithelial cells indicating antigen presentation and checkpoint in tissues from high and low proliferation TNBC tumors in MIBI cohort. c-e. Kruskal-Wallis H-test P-value given in panel title. Post-hoc Tukey HSD used for pairwise comparisons between groups. F-G. P-value from Mann-Whitney U rank test given in panel title.

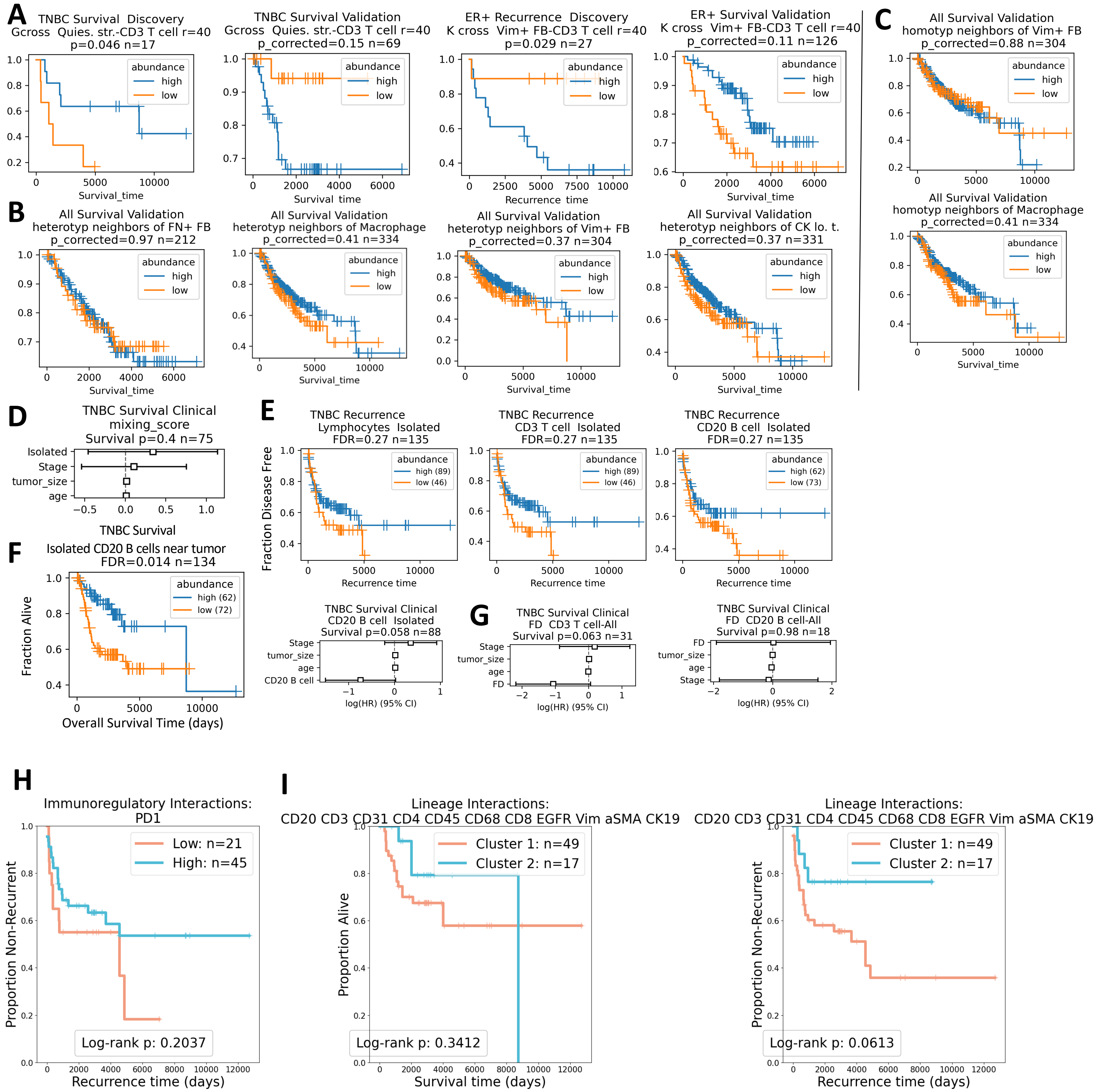

S11. Prognostic value of tumor immune spatial metrics

A. Kaplan-Meier (K-M) analysis showing opposite overall survival (OS) associations in discovery vs validation cohorts for quiescent stroma to T cell Gcross function in TNBC (left panels) and Vimentin (Vim)+ fibroblast (FB) to T cell K cross function in ER+ (right panels), 75 µm radius. B. K-M estimate of OS stratified by of heterotypic neighbors of fibronectin (FN)+ FB, macrophage, Vim+ FB and cytokeratin low tumor, all subtypes C. K-M estimate of OS stratified by of homotypic neighbors of macrophage and Vim+ FB, all subtypes. D. Multivariate CPH modelling of mixing score and clinical co-variates versus OS in TNBC. E. K-M estimate of recurrence-free survival (RFS) in TNBC stratified by isolated lymphocytes, CD3 T cells and CD20 B cells. F. K-M estimate of OS (left) and multivariate CPH (right) for isolated B cells near tumor. G. Multivariate CPH modelling of fractal dimension difference of T (left) and B lymphocytes (right) and clinical co-variates versus OS in TNBC. H. K-M estimate of RFS vs. PD-1 interactions in CycIF TNBC. I. K-M estimate of OS vs. lineage marker interactions in CycIF TNBC. A-C, E-F, H-I. Kaplan-Meier survival curves. p-value from log-rank test and n number of patients given in panel title and/or legend. y-axis is fraction of patients alive or recurrence free and x-axis survival/recurrence time is in days. D, F, G. CPH p-value for spatial metric and n number or patients in panel title. H-I. markers used in analysis in K-M panel title.

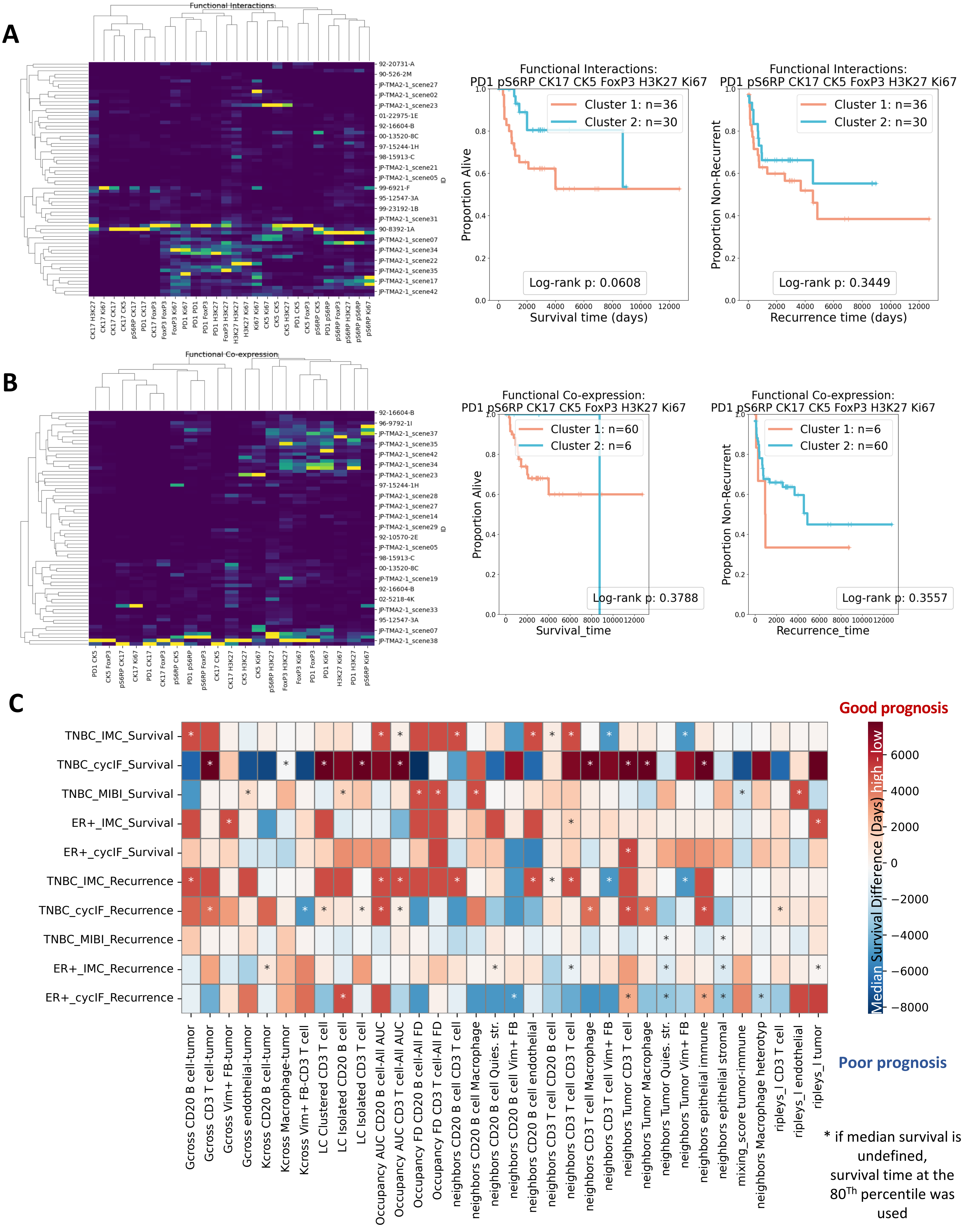

S12. Prognostic value of immunoregulatory, lineage and functional interactions, co-expression.

A. Kaplan-Meier (K-M) analysis of recurrence free survival (RFS) vs. PD-1 interactions in CycIF TNBC. B. K-M analysis of lineage marker interactions vs. overall survival (OS) in CycIF TNBC. C. Clustered heatmap of total functional interactions in each patient (left), K-M of OS (center) and RFS (right) for the two clusters from heatmap. D. Clustered heatmap of total functional co-expression in each patient (left), K-M of OS (center) and RFS (right) for the two clusters from heatmap. A-B. markers used in analysis in K-M panel title, p-value from log-rank test, n number of patients in panel legend.

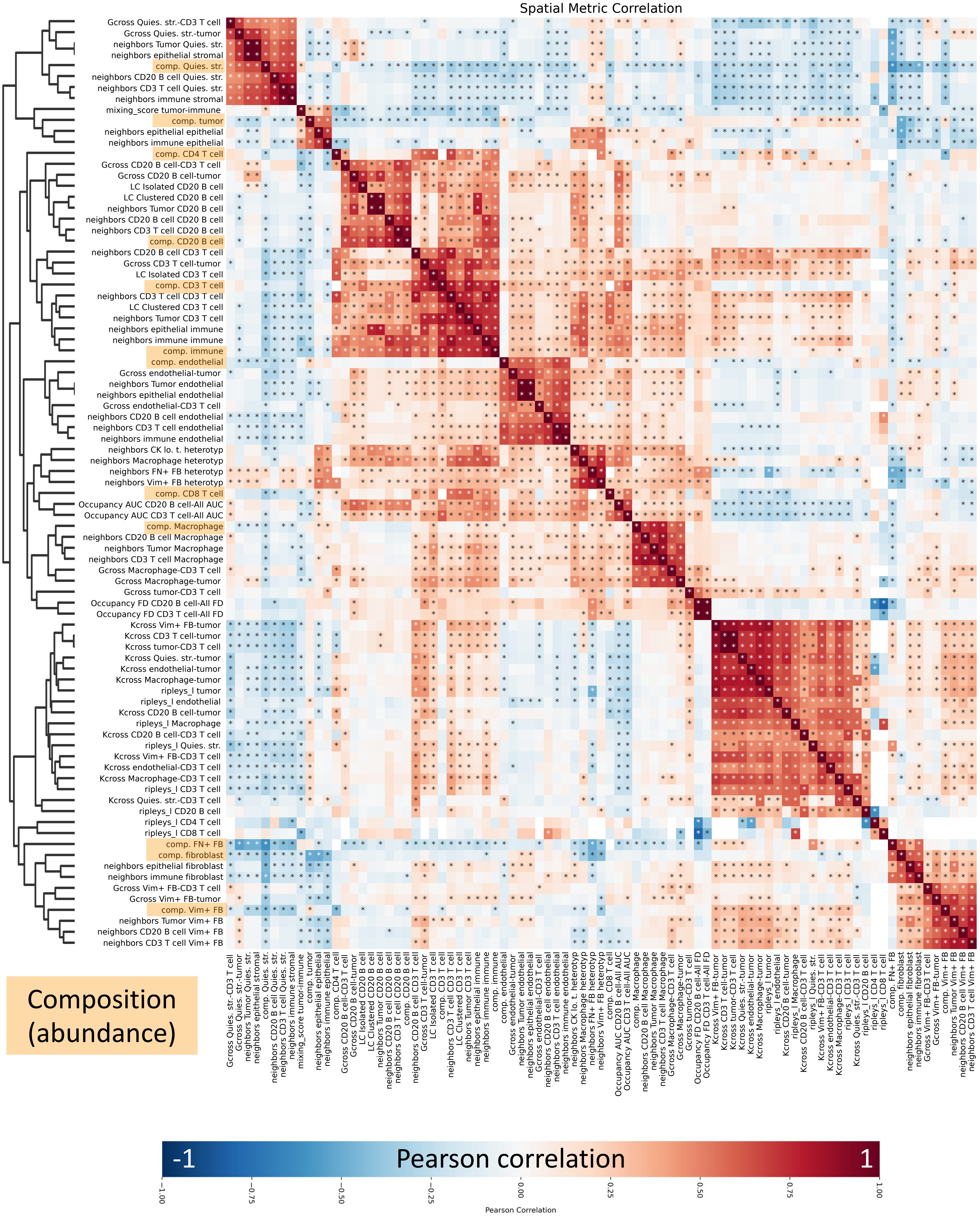

S13. Correlation of Spatial Metrics and Tissue Composition.

Heatmap of Pearson correlation between spatial metrics and tissue cell type composition (fraction of cells in tissue). Composition (comp.) variables are highlighted in orange. Asterisk denotes significant correlation ($p<0.05$). Dendrogram shows hierarchical clustering of metrics. N=344 patients for celltypes shared across three platforms.

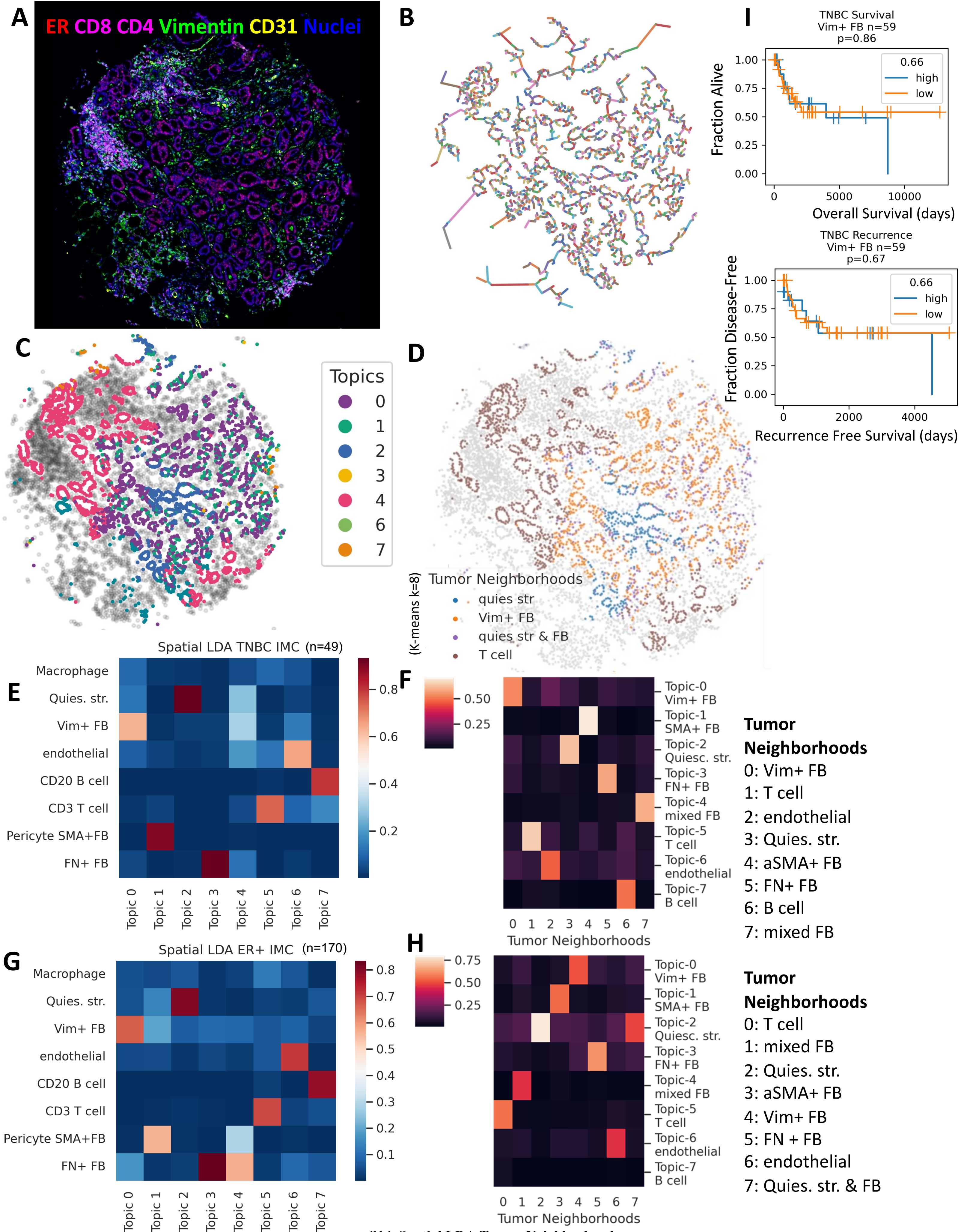

S14. Spatial LDA Tumor Neighborhoods.

A. CyCIF staining of tissue showing estrogen-receptor+ tumor (ER), T cell (CD4 and CD8) fibroblast (vimentin) and endothelial (CD31) markers. B. Tumor cells from (A) nearest neighbor map for calculation of a spatial parameter to increase the likelihood that adjacent cells share the same topics. C. Tumor cells from (A) colored by the highest weighted spatial latent Dirichlet allocation (LDA) topic. D. Tissue from (A) with tumor cells colored by their spatial LDA neighborhood cluster. Tumor cells colored by T cell- (brown), quiescent stroma- (blue), mixed fibroblast- (purple) and vimentin+ fibroblast-neighborhoods (orange) defined by k-means clustering (K=8) of the single-cell topic matrix. E. Heatmap of stromal cell enrichment in spatial LDA topics of 100 µm tumor neighborhoods in TNBC tissue from the IMC platform. F. Heatmap of fraction of each topic in each neighborhood cluster resulting from K-means clustering (k=8) of spatial LDA topics from (E). G-H. heatmaps as defined in E and F, for ER+ tumors from the IMC cohort. I. Kaplan-Meier estimate of OS (top) and RFS (bottom) in TNBC, stratified by Vim+ FB abundance. E, G. N number of patients in LDA model shown in panel title.

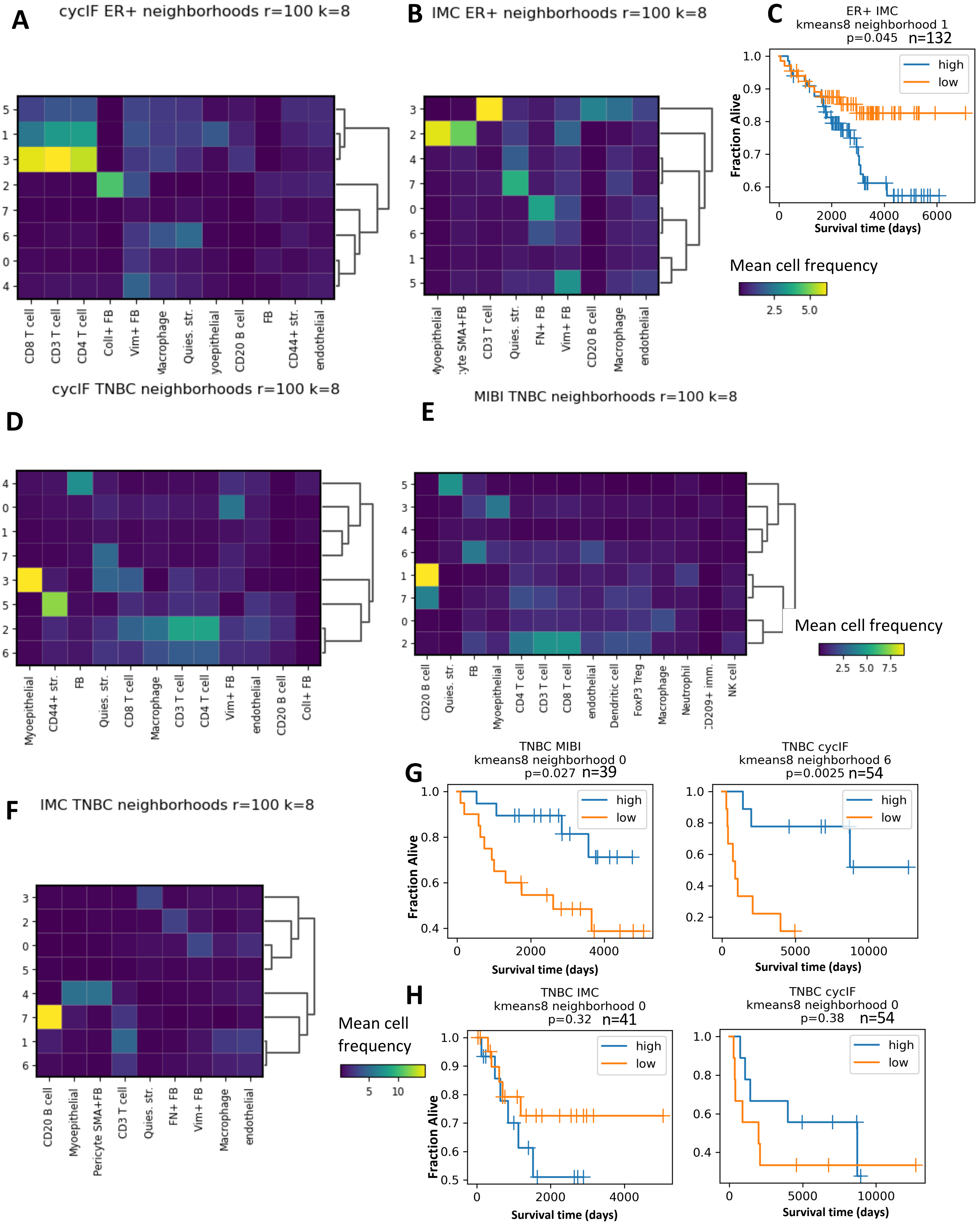

S15. Neighborhoods defined by directly Kmeans clustering the cell counts.

A-B. Heat map of mean stromal cell frequency in neighborhood clusters in ER+ breast cancer tissues. Stromal cell counts in a 100 um radius surrounding epithelial cells were clustered with the kmeans algorithm, k=8, for the CyCIF (a) and IMC cohort (B). C. Kaplan-Meier analysis of a neighborhood from IMC ER+ breast cancer (A-B) that was significantly associated with overall survival. D-F. Heat map of mean stromal cell frequency in neighborhood clusters in TNBC tissues from CyCIF (D), MIBI (E) and IMC cohort (F). G. Kaplan-Meier analysis of neighborhoods from TNBC (D-E) that were significantly associated with overall survival. H. Kaplan-Meier analysis of Vim+ FB neighborhoods in TNBC versus overall survival. C, G-H. High and low neighborhood frequency defined by the median of the cohort. p-values from log-rank test and n number of patients given in panel title.

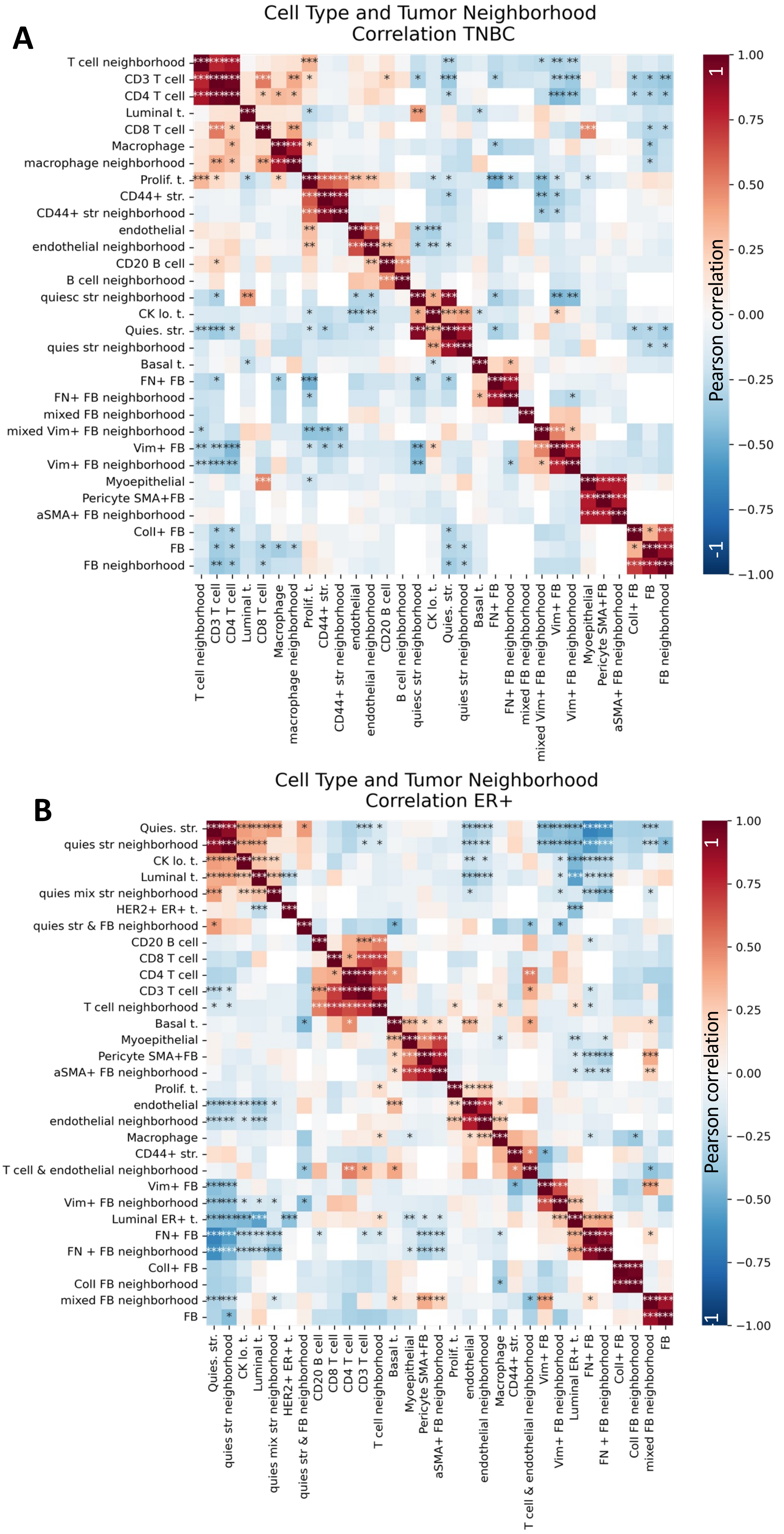

S16. Correlation of tumor neighborhoods and tissue composition.

A. Heatmap of Pearson correlation between spatial LDA neighborhoods and fraction of cells in tissue in TNBC from combined CyCIF and IMC cohorts. Neighborhood variables are labelled as such and composition variables are just the cell type label. Asterisk denotes significant correlation (*p<0.05, **p<0.005, ***p<0.001). N=106 patients. B. Heatmap of Pearson correlation between spatial LDA neighborhoods and fraction of cells in tissue in ER+ tumors from combined CyCIF and IMC cohorts, labelled as in (A). N=200 patients.

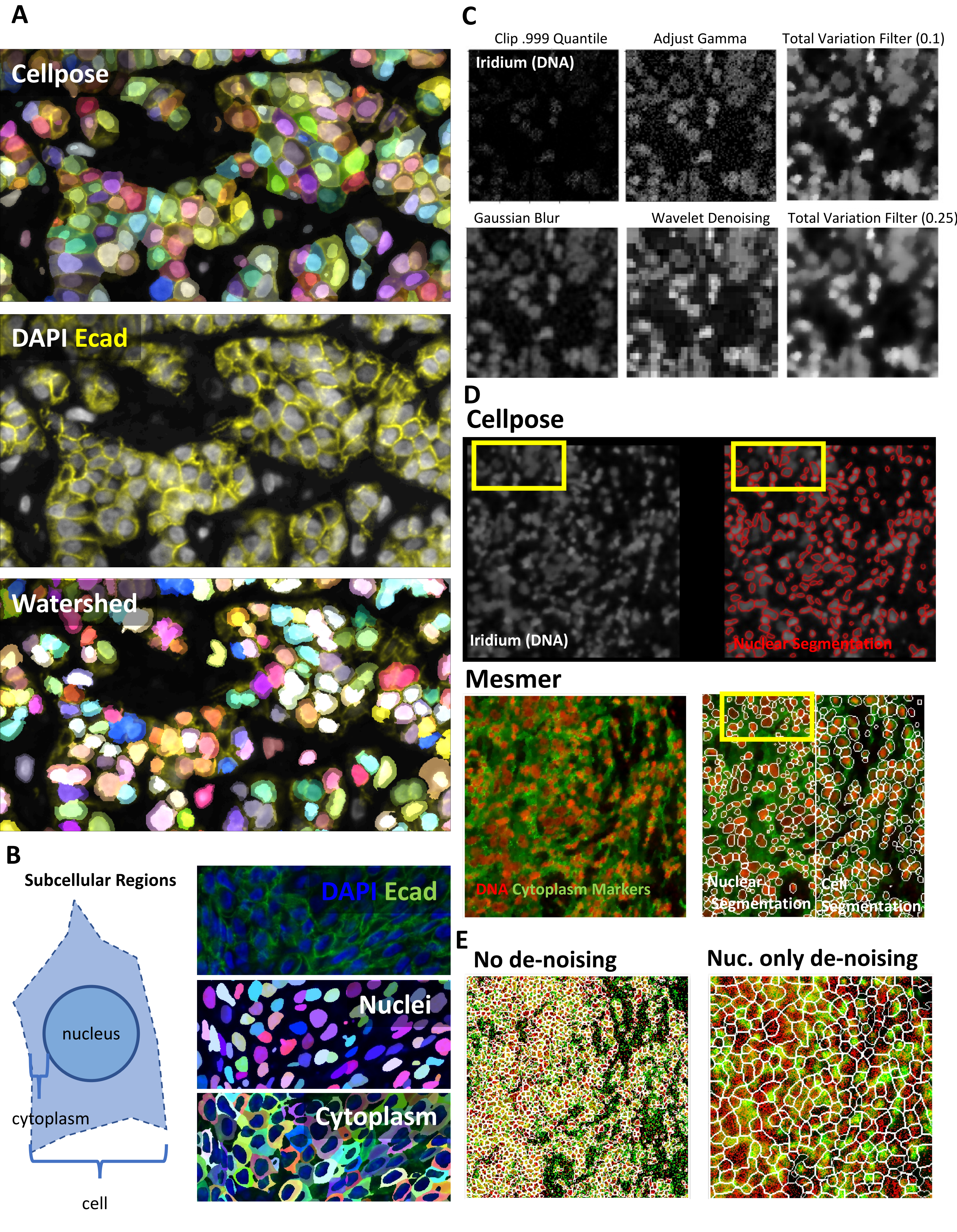

S17. De-noising and Segmentation Optimization.

A. CyCIF Cellpose nuclear and cell segmentation (top) versus watershed segmentation (bottom). B. Construction of cytoplasm mask from cell and nuclear masks after matching with mplexable. C. Image processing steps tested for the IMC denoising pipeline. D. IMC Cellpose segmentation (top) versus Mesmer segmentation (bottom). E. IMC Mesmer segmentation of gamma-adjusted only image of ROI shown in (D) (left), and of de-noised nuclear channel with no cytoplasm denoising (right).

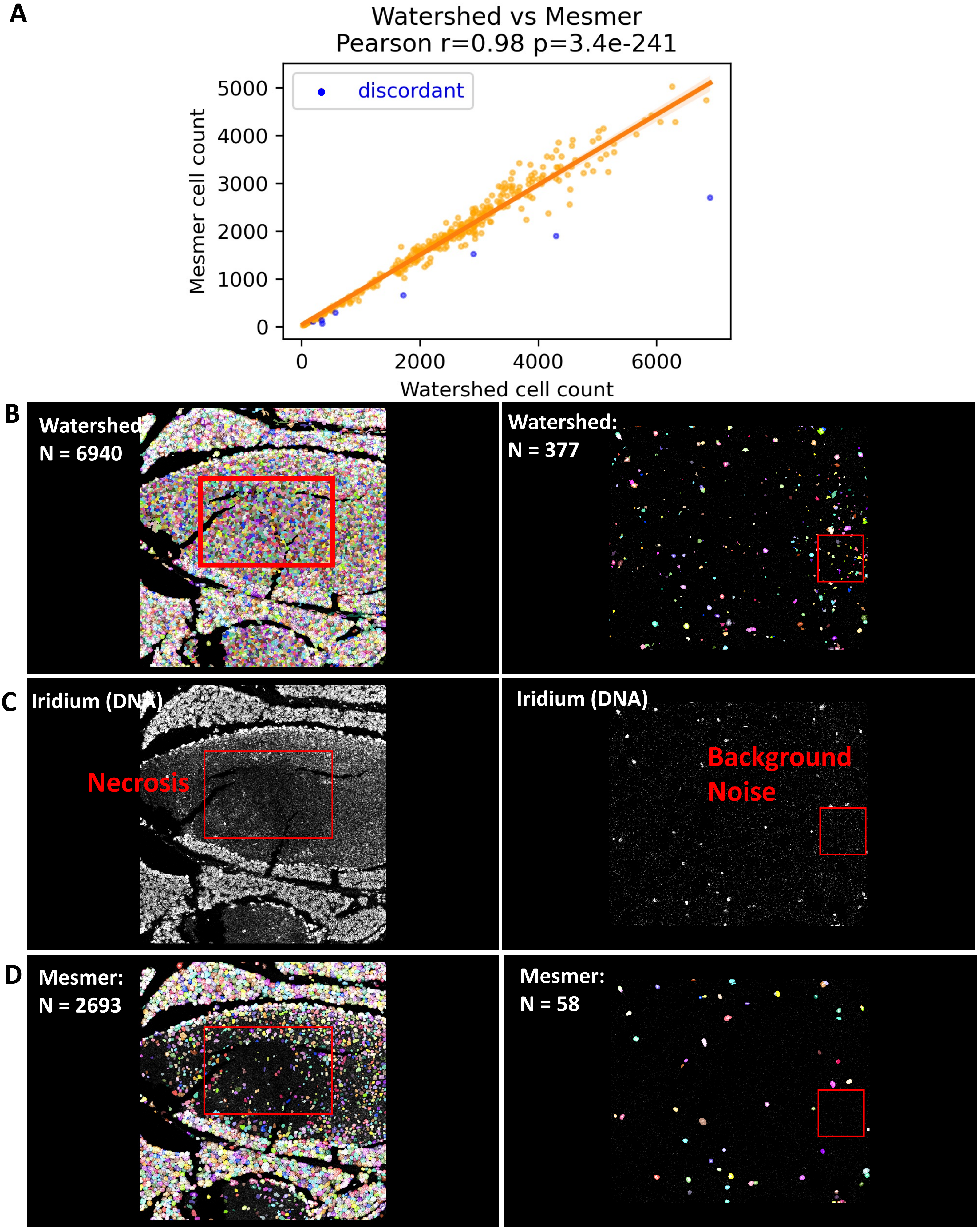

S18. Mesmer versus Watershed Segmentation.

A. Pearson correlation between cell counts of watershed versus Mesmer segmentation in IMC tissues. Tissues with >50% change shown in blue. B. Watershed segmentation of two selected tissues with discordant cell counts between segmentation methods. Red boxes indicate areas of over segmentation. C. DNA channel of selected tissues. D. Mesmer segmentation of selected tissues. B, D. N number of cells segmented shown on panel.

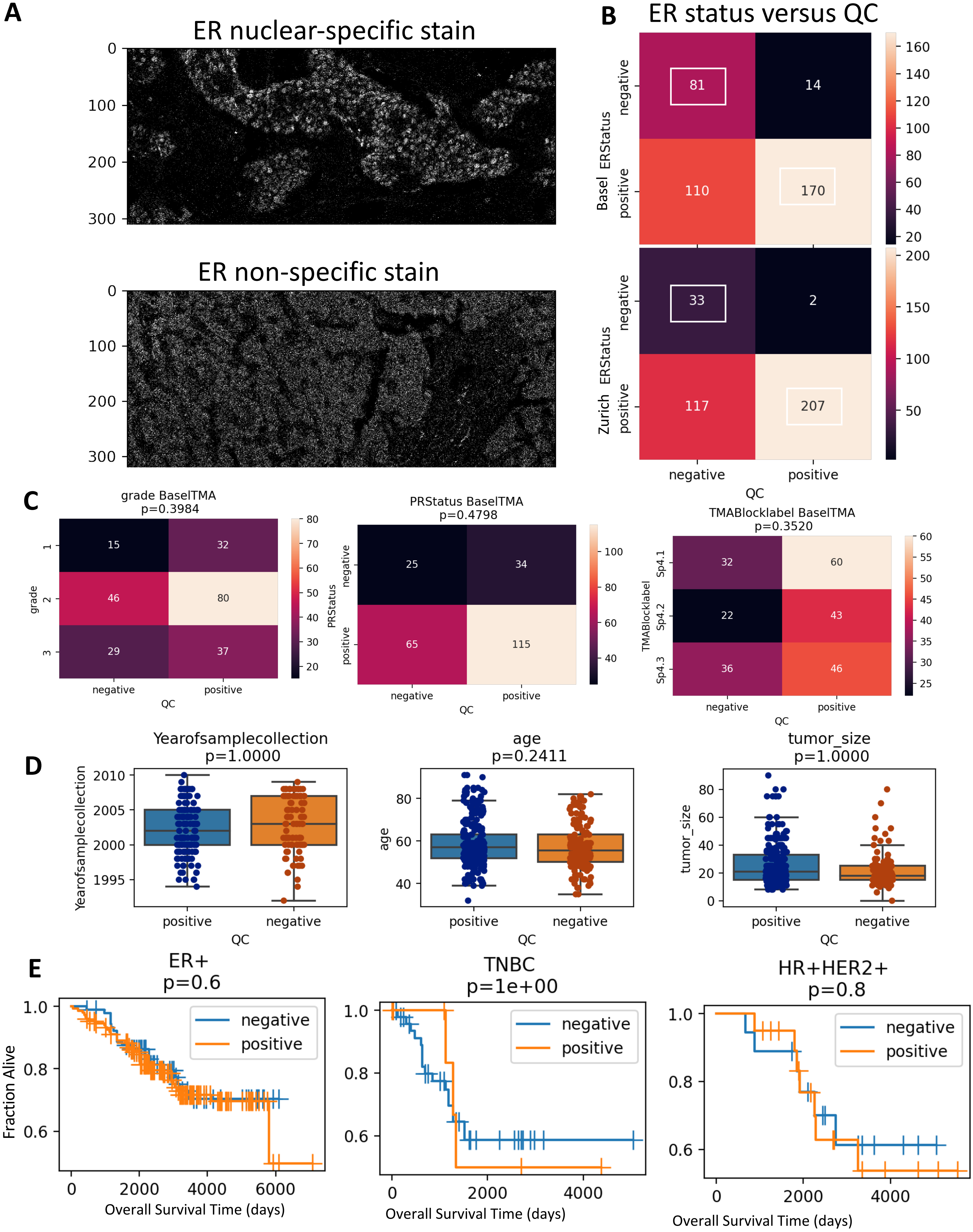

S19. ER Quality Control.

A. Representative IMC images of estrogen receptor channel QC images sorted for positive (top) and negative (bottom) nuclear ER staining. B. Image ROIs classified by annotated ER status (y-axis) versus our QC call for ER positive or negative (x-axis) in two IMC TMAs. Samples on the diagonal (white boxes) were used. C. Grade, PR status and TMA block versus QC calls, p-values from Chi-squared analysis shown in panel title. B-C. N number of ROIs in each category annotated on heatmap cells. D. Year of sample collection, patient age and tumor size versus QC status of each ROI, p-values for Wilcoxon-rank sum test shown in panel title. E. Kaplan-Meier curves for OS versus QC status, p-values from log-rank test.

A

CycIF			
**Antigen**	**Description**	**Antigen**	**Description**
**Cytokeratins**		**Immune Context**	
CK5	Basal CK	CD45	pan-Immune
CK7	Luminal CK	CD3	T Cells
CK8	Luminal CK	CD20	B Cells
CK14	Basal CK	CD8	Cytotoxic T cell
CK17	Basal CK	CD4	Helper T cell
CK19	Luminal CK	CD68	Macrophage
**Adhesion Molecules**		PD1	T cell exhaustion
Ecadherin	Cell Adhesion	FoxP3	Regulatory T cell
CD44	Cell Adhesion	GRNZB	Cytotoxicity
**Hormone Receptors**		**Hypoxia and Metabolism**	
ER	Estrogen receptor	HIF1a	Hypoxia
PR	Progesterone Receptor	Glut1	Glucose Transport
AR	Androgen Receptor	CoxIV	Mitochondria
**RTK Signaling**		**Cell Growth and Division**	
HER2	RTK	Ki67	Proliferation
EGFR	RTK	PCNA	Proliferation
**Endothelial**		p-HH3	Mitosis
CD31	Endothelial	p-S6	Growth
PDPN	Lymphatic Endothelial	p-RB	Cell Division
**Stromal Markers**		**Nuclear**	
SMA	Myoepithelial cells	Lamin AC	Nuclear Membrane
Vimentin	Mesenchymal Cells	Lamin B1	Nuclear Membrane
Collagen IV	Basement Membrane	Lamin B2	Nuclear Membrane
Collagen I	Extracellular Matrix	**Cell Death**	
**Epigenetic Mark**		cleaved PARP	Apoptosis
H3K27	Gene Repression		
H3K4	Gene Accessibility		

MIBI			
**Antigen**	**Description**	**Antigen**	**Description**
**Cytokeratins**		**Immune Context**	
CK6	Basal CK	CD45	pan-Immune
CK17	Basal CK	CD3	T Cells
pan-CK	Luminal & Basal	CD20	B Cells
**Adhesion Molecules**		CD8	Cytotoxic T cell
Beta catenin	Cell Adhesion	CD4	Helper T cell
**Transcription Factors**		CD68	Macrophage
p53	Tumor suppressor	PD1	Immune regulation
**RTK Signaling**		FoxP3	Regulatory T cell
EGFR	RTK	CD45RO	Memory T cells
**Endothelial**		CD56	NK cells
CD31	Endothelial	HLA Class 1	Antigen Presentation
**Stromal Markers**		HLA DR	Antigen Presentation
SMA	Myoepithelial cells	CD11c	Dendritic cells
Vimentin	Mesenchymal Cells	CD11b	pan-Myeloid
**Epigenetic Mark**		CD138	Plasma cells
H3K27me3	Gene Repression	CD16	NK cells
H3K9ac	Gene Accessibility	CD209	Antigen Presentation
**Cell Growth and Division**		IDO	Immune regulation
Ki67	Proliferation	Lag3	Immune regulation
p-S6	Growth	MPO	Neutrophils
		PDL1	Immune regulation
		CD63	Monocytes

IMC			
**Antigen**	**Description**	**Antigen**	**Description**
**Cytokeratins**		**Immune Context**	
CK5	Basal CK	CD45	pan-Immune
CK7	Luminal CK	CD3	T Cells
CK8/18	Luminal CK	CD20	B Cells
CK14	Basal CK	CD68	Macrophage
CK19	Luminal CK	**Transcription Factors**	
AE1/AE3	Pan-CK	p53	Tumor Suppressor
**Adhesion Molecules**		cMyc	Proto-Oncogene
E/P-cadherin	Cell Adhesion	GATA3	Luminal TF
CD44	Cell Adhesion	Twist	EMT TF
**Hormone Receptors**		Slug	EMT TF
ER	Estrogen receptor	**Hypoxia and Metabolism**	
PR	Progesterone Receptor	HIF1a	Hypoxia
**RTK Signaling**		Glut1	Glucose Transport
HER2	RTK	CoxIV	Mitochondria
HER2	RTK	**Cell Growth and Division**	
**Endothelial**		Ki67	Proliferation
vWF	Endothelial	p-HH3	Mitosis
CD31	Endothelial	p-S6	Growth
**Stromal Markers**		P-MTOR	Growth
Fibronectin	Matrix Glycoprotein	**Nuclear**	
SMA	Myoepithelial	Histone H3	Chromatin
Vimentin	Mesenchymal	**Cell Death**	
**Epigenetic Mark**		cleaved PARP	Apoptosis
H3K27	Gene Repression	cleaved Casp3	Apoptosis

B

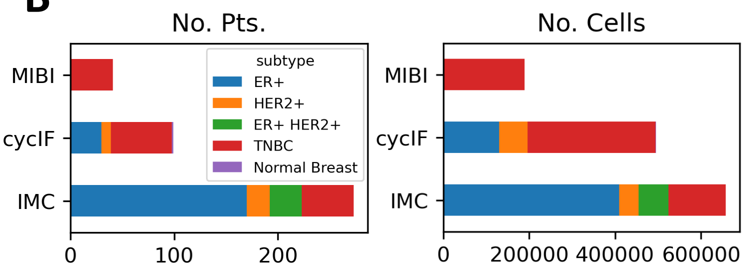

C

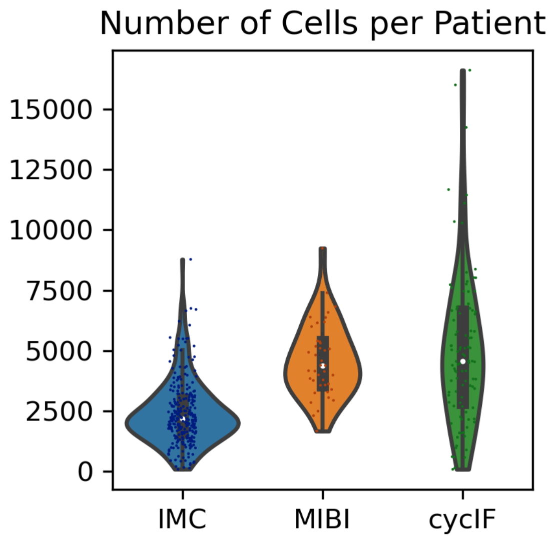

S20. Comparison of breast cancer MXI panels and dataset sizes.

A. The antibody panels from the three breast cancer datasets: our own CyCIF data from two breast cancer TMAs and publicly available IMC and MIBI data, including markers for cytokeratins, adhesion molecules, hormone receptors, receptor tyrosine kinase (RTK) signaling, cell growth and division, endothelial, immune and stromal cells. B. Number of patients (left) and cells (right) in each dataset, colored by subtype. C. Number of cells per patient in each dataset. Box shows the median and interquartile range, whiskers show the 95% confidence intervals.
